# Single-cell analysis and stochastic modelling unveil large cell-to-cell variability in influenza A virus infection

**DOI:** 10.1038/ncomms9938

**Published:** 2015-11-20

**Authors:** Frank S. Heldt, Sascha Y. Kupke, Sebastian Dorl, Udo Reichl, Timo Frensing

**Affiliations:** 1 Department of Bioprocess Engineering, Max Planck Institute for Dynamics of Complex Technical Systems, Sandtorstrasse 1, 39106 Magdeburg, Germany; 2 Chair of Bioprocess Engineering, Otto von Guericke University Magdeburg, Universitaetsplatz 2, 39106 Magdeburg, Germany

## Abstract

Biochemical reactions are subject to stochastic fluctuations that can give rise to cell-to-cell variability. Yet, how this variability affects viral infections, which themselves involve noisy reactions, remains largely elusive. Here we present single-cell experiments and stochastic simulations that reveal a large heterogeneity between influenza A virus (IAV)-infected cells. In particular, experimental data show that progeny virus titres range from 1 to 970 plaque-forming units and intracellular viral RNA (vRNA) levels span three orders of magnitude. Moreover, the segmentation of IAV genomes seems to increase the susceptibility of their replication to noise, since the level of different genome segments can vary substantially within a cell. In addition, simulations suggest that the abortion of virus entry and random degradation of vRNAs can result in a large fraction of non-productive cells after single-hit infection. These results challenge current beliefs that cell population measurements and deterministic simulations are an accurate representation of viral infections.

## Abstract (web summary)

Cell-to-cell variability in viral infection means that cell population measurements may not be an accurate representation of the process. Using both experimental and modelling approaches the authors confirm this notion showing that influenza virus infections are variable processes affected by intrinsic and extrinsic noise.

## Graphical abstract

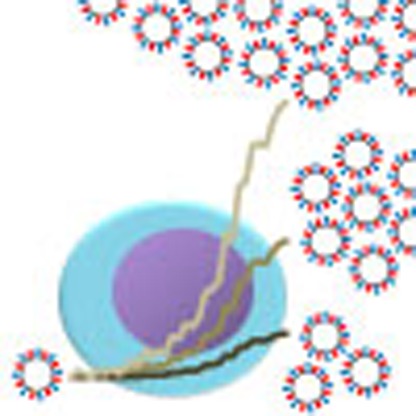

Viral infections can be initiated by a small number of infectious particles or even a single virion. In these cases, successful replication of the virus relies on reactions that comprise very few molecules (for example, a single copy of the viral genome and a handful of proteins). Such reactions are, however, subject to stochastic fluctuations inherent to all molecular processes, which can cause large cell-to-cell heterogeneity. Moreover, individual host cells may differ in basic properties such as their protein content or cell cycle stage introducing additional variation in the cell population. These differences between cells can have important consequences for virus replication. For instance, noise in viral protein expression during HIV replication has been suggested to lead to a small subpopulation of latent cells, which are difficult to target pharmacologically^1^. Such subpopulations may contribute disproportionally to virus spread and persistence in the long term.

One of the first studies on cell-to-cell variability in viral infection was conducted by Max Delbrück in the 1940s using phage-infected *Escherichia coli*^2^. In these experiments, the burst size of cells (that is, the number of progeny virions released per cell) varied significantly from below 20 to over 1,000 phages revealing a surprisingly broad distribution of virus yields. Later, similar results were obtained for mammalian cells infected by the vesicular stomatitis virus (VSV), which show differences in cell-specific virus titres that span over 300-fold^3^,^4^. In general, the cell-to-cell variation in a particular molecule's abundance comprises two parts: (i) stochastic fluctuations inherent to the biochemical reactions involved in the turnover of the molecule, which are known as intrinsic noise, and (ii) fluctuations in the amount of other cellular components influencing these biochemical reactions, that is, extrinsic noise^5^. Viruses rely on cellular factors and it has been shown for adherent cells that the cell's population context (for example, whether it originates from a sparsely or densely populated area of a culture flask) causes cell-to-cell heterogeneity in their susceptibility to infections^6^. Moreover, cellular characteristics such as the cell size and cell cycle stage were found to cause some of the titre fluctuations observed between VSV-infected cells^3^. For the same virus, mathematical modelling suggests that stochastic viral gene expression (that is, intrinsic noise) can lead to cell-to-cell variability^7^. Yet, these theoretical predictions have not been validated experimentally. Recently, Schulte *et al.* investigated poliovirus infection at the single-cell level using two multiplicities of infection (MOIs) and, again, found a wide spread in virus titres^8^. Moreover, they show that intracellular viral RNA (vRNA) levels can span one to two orders of magnitude. Surprisingly, however, poliovirus yields were not correlated to these RNA levels at high MOI.

So far, single-cell analysis has mainly focused on viruses that possess a single molecule of genomic information such as poliovirus or VSV. Yet, noise may have an even greater effect on segmented genomes, since the copy number of individual viral genes can vary independently during their replication introducing additional heterogeneity between the infected cells. Here, we investigate influenza A virus (IAV), a segmented virus and important human pathogen that causes annual epidemics and occasionally severe pandemics. In particular, we focus on an infection of Madin–Darby canine kidney (MDCK) cells with influenza virus A/Puerto Rico/8/34 (PR8) of the H1N1 subtype, a prototype experimental system for IAVs that is also widely used in cell culture-based vaccine production^9^,^10^. Studying the replication of a segmented virus such as IAV provides the opportunity to distinguish between intrinsic and extrinsic noise by measuring the RNA levels of different genome segments in individual cells. A similar experimental approach has been used by Elowitz *et al.* to analyse the source of noise in gene expression in *E. coli*^5^.

The genome of IAV comprises eight segments each encoding at least one viral protein^11^. Influenza vRNAs are single-stranded and of negative polarity. Following virus entry, they move into the nucleus of a host cell where they engage in viral transcription and produce a positive-strand copy of the genome, the complementary RNA (cRNA)^12^. Newly produced cRNA then serves as a template for progeny vRNAs, which can participate in messenger RNA (mRNA) and cRNA synthesis or leave the nucleus to form progeny virions. During infection, most vRNAs and cRNAs do not exist as naked RNA but rather form viral ribonucleoproteins (vRNPs and cRNPs, respectively)^13^. Each RNP consists of an RNA template, a tripartite viral polymerase complex and multiple copies of the nucleoprotein (NP). It, thus, represents an independent functional unit of influenza virus replication and a potential source of noise.

To quantify the cell-to-cell variability in IAV infection, we developed an experimental approach that facilitates the isolation of infected cells into individual wells of a culture plate. We subjected the supernatant of these cells to a plaque assay to determine the amount of infectious virus progeny, and performed real-time reverse transcription quantitative PCR (RT–qPCR) on cell lysates to quantify the absolute number of intracellular vRNAs of different genome segments. Both assays revealed a substantial cell-to-cell heterogeneity. To interpret the results systematically, we also derived a stochastic mathematical model of the intracellular viral life cycle. This model allows us to simulate the extent to which molecular noise affects single-cell IAV infections and to study infection scenarios that cannot be measured directly. In combination, our experimental data and simulations show that the variability in vRNA levels is caused by both, extrinsic noise and stochastic fluctuations intrinsic to vRNA synthesis. Furthermore, we demonstrate that differences in intracellular vRNA content are one reason for the large cell-to-cell heterogeneity in IAV titres observed in our experiments. Finally, we present simulations that indicate that single-hit infections, where only a single virion enters the cell, predominantly fail to produce progeny virions due to stochastic effects. Together, these results shed light on the variability of IAV infection at the single-cell level, which may play an important role during the early stages of an infection, where the virus establishes itself in a new host.

## Results

### Single-cell analysis reveals large variability in IAV titres

**Figure 1:**
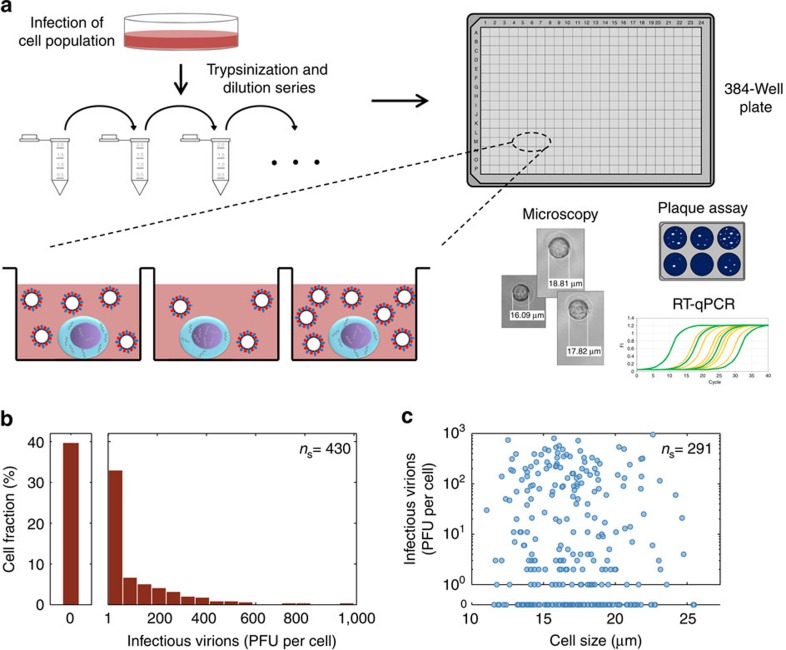
Single-cell analysis approach and virus yields of IAV-infected cells. (**a**) Scheme of the experimental procedure. A population of adherent MDCK cells was infected with influenza PR8 virus at an MOI of 10, incubated and afterwards trypsinized to obtain a cell suspension. Subsequently, the diluted cell suspension was transferred to a 384-well plate and wells containing single cells were identified by phase-contrast microscopy. At 12 h.p.i., virus titres in the supernatant were determined by the plaque assay and intracellular vRNAs were quantified by real-time RT–qPCR. (**b**) Distribution of virus yield. The first bar on the left of the histogram indicates the fraction of cells that show no virus release (0 PFU). Illustration includes pooled data of multiple independent experiments (*n*=8). (**c**) Correlation between virus titre and cell size. The pooled results of multiple independent experiments (*n*=4) are depicted. *n*_s_ indicates the number of single cells analysed.

To study the heterogeneity in IAV replication, we performed a single-cell analysis of infected cells (Fig. 1a). First, a population of MDCK cells was infected with PR8 virus at an MOI of 10. Here, the high MOI ensures that all cells are infected simultaneously. At 2.5 h post infection (h.p.i.), the cells were trypsinized, serially diluted and transferred to a 384-well plate, such that each well would receive on average one cell. Wells containing single cells were identified by phase-contrast microscopy. Consistent with the Poisson distribution, we found that ≈35% of the wells contained a single cell. To determine the virus yield of these single cells, their complete supernatant was subjected to a plaque assay at 12 h.p.i. Moreover, a cell lysis step facilitated the analysis of intracellular vRNAs by real-time RT–qPCR. To confirm that the experimental procedure did not interfere with virus replication, we compared single-cell- and population-derived measurements. For both approaches, cell-specific virus titres and intracellular vRNA levels were in the same order of magnitude (Supplementary Fig. 1).

Our experiments reveal a large heterogeneity in the productivity of individual infected cells with an almost 1,000-fold difference in virus titres (Fig. 1b). This variability, by far, exceeds the technical noise of the plaque assay, which shows a mean relative s.d. of 18% (Supplementary Fig. 2a). In particular, most productive cells released only 1–75 plaque-forming units (PFUs). However, we also detected cells that yielded several hundred PFUs. Moreover, roughly 40% of the infections did not produce a plaque titre although these cells were tested positive for vRNAs, which confirms their infection (Supplementary Fig. 3). As virus replication relies on the biosynthetic machinery of a host cell and large cells may harbour an increased amount of cellular resources, we also studied whether the synthesis of progeny virions depends on the cell size (Fig. 1c). However, we could not find a correlation between cell diameter and virus yield. Taken together, our single-cell assay reveals a large heterogeneity in virus titres, which cannot be attributed to differences in cell size.

### Noise in vRNAs may cause heterogeneity in virus titres

**Figure 2:**
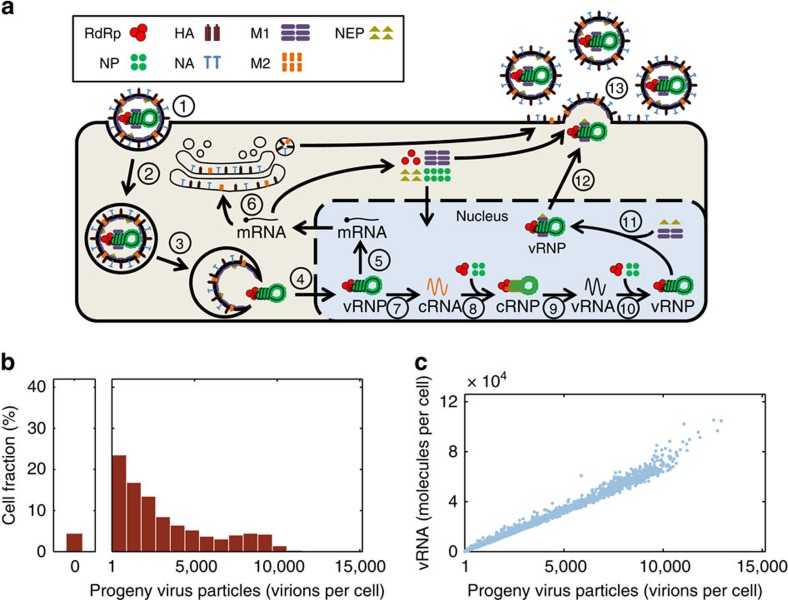
Stochastic simulation of IAV replication. (**a**) Schematic depiction of the model. Different steps are assigned by numbers: 1, attachment; 2, endocytosis; 3, fusion with late endosomes; 4, nuclear import of vRNPs; 5, viral mRNA transcription; 6, protein translation; 7, replication (cRNA synthesis); 8, cRNA encapsidation; 9, replication (vRNA synthesis); 10, vRNA encapsidation; 11, binding of matrix protein 1 (M1) and nuclear export protein (NEP); 12, nuclear export; 13, virus assembly and budding. HA, haemagglutinin; M2, matrix protein 2; NA, neuraminidase; RdRp, viral RNA-dependent RNA polymerase. (**b**) Number of progeny virions released by individual infected cells until 12 h.p.i. for an infection at an MOI of 10. (**c**) Correlation between the vRNA level of the least-abundant genome segment in a cell at 12 h.p.i. and the number of progeny virions produced by that cell for an infection at an MOI of 10.

To elucidate the source of the high variability in virus yields, we developed a novel stochastic mathematical model of IAV replication (Fig. 2a). The model comprises key steps of the intracellular viral life cycle from virus entry to budding. It extends a previously published deterministic description, that captures a variety of experimental data including the cell population averages of vRNAs^14^,^15^, by considering the inherently random nature of biochemical reactions. In agreement with the measurements, the simulation of 3,000 cells with the stochastic model showed a high variability in virus titres and a bias towards low productivity (Fig. 2b). However, in contrast to the experimental data, only 4% of the cells in our model are non-productive. In this context, it has to be considered that the model provides the total number of released progeny viral particles, which includes infectious as well as noninfectious viruses. Hence, we cannot compare the experimental data quantitatively with the simulations.

In the established model, viral proteins are present in excess and cell-specific virus yields depend primarily on the abundance of vRNAs available for packaging. More precisely, the productivity of an infected cell is proportional to the level of the least-abundant genome segment in that cell (Fig. 2c). Thus, we conclude from our model that noise in intracellular vRNA levels may cause the majority of the observed cell-to-cell variability in virus titres.

### Extrinsic and intrinsic noise affect virus replication

**Figure 3:**
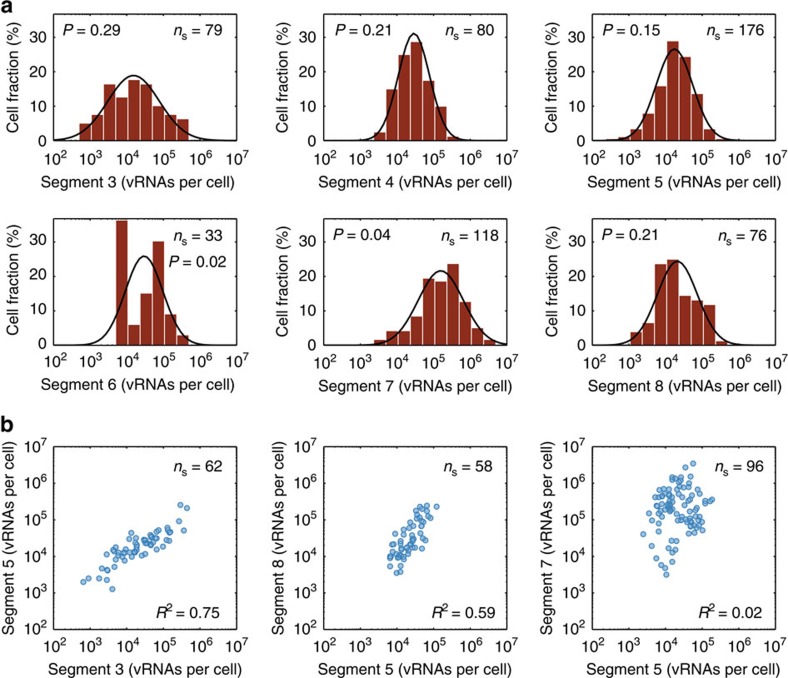
Distributions of vRNA levels between infected single cells and correlation of vRNA segments. Cells were infected at an MOI of 10 and analysed for their intracellular vRNA content at 12 h.p.i. via real-time RT–qPCR. *n*_S_ indicates the number of single cells analysed. (**a**) Frequency distributions of vRNA levels of segments 3–8. The illustrations comprise pooled data of multiple independent experiments (*n*=2 for segment 3, 4 and 7; *n*=5 for segment 5; *n*=1 for segment 6; *n*=3 for segment 7). The solid lines describe log-normal distributions fitted to the data. The *P* value from Shapiro–Wilk normality test is indicated. (**b**) Intersegment dependencies of vRNAs. The illustrations comprise pooled data of multiple independent experiments (*n*=2 for segment 3 and 5; *n*=2 for segment 5 and 8; *n*=3 for segments 5 and 7). The coefficient of determination (*R*^2^) is provided.

To study the heterogeneity in vRNA abundance experimentally, we performed real-time RT–qPCR on single infected cells. Indeed, these measurements reveal a high variability in virus replication with intracellular vRNA levels spanning up to three orders of magnitude (Fig. 3a). This cell-to-cell variability is significantly higher than the technical error of the real-time RT–qPCR, which shows a mean relative s.d. of 27% (Supplementary Fig. 2b). In addition, the vRNA levels of segments 3–5 and 8 were log-normally distributed confirmed by the Shapiro–Wilk normality test (indicated by *P*≥0.05, considering a significance level of *α*=5%). Yet, for segments 6 and 7 no such distribution was present. In the case of segment 6, this may be due to the low number of measurements, as increasing the sample size by pooling additional experiments can improve the agreement with a log-normal distribution (Supplementary Fig. 4a,b). A potential explanation for segment 7 is provided in the discussion. Also note that the mean vRNA levels from independent experiments were similar, suggesting that our single-cell measurements are highly reproducible and that we can pool the data sets to obtain a higher sample size (Supplementary Fig. 4c).

Next, we simultaneously quantified the amount of vRNAs of up to three different genome segments in single infected cells to investigate their dependency (Fig. 3b; Supplementary Fig. 5). We observe a positive correlation between the abundances of most genome segments, with the exception of segment 7, which is not correlated to any other segment. A positive intersegment correlation can be caused by noise sources extrinsic to virus replication such as fluctuations in cellular factors. Typically, these factors would affect the level of all individual vRNAs in a cell evenly and would, hence, increase their correlation. Besides extrinsic noise, the inherent randomness of biochemical reactions can create intrinsic noise, which affects the number of vRNAs in a single cell differently, and results in a decrease in their correlation. We observed such intrinsic noise in IAV replication, manifested by the deviation of vRNA levels from the ideal correlation between two genome segments. The observed variability in vRNA synthesis is neither caused by genetic heterogeneity of the virus population nor by the presence of so-called defective interfering particles (DIPs). We could demonstrate this by using a triple plaque-purified seed virus. The plaque purification reduces the genetic heterogeneity and an isolate was chosen that concurrently showed no detectable amounts of DIPs. Still, infections with this isolate resulted in the same cell-to-cell variability in intracellular vRNA levels (Supplementary Figs 6 and 11). In summary, by quantifying different genome segments within individual cells, our experiments demonstrate that both extrinsic as well as intrinsic noise contribute to the high variability in IAV replication.

### Noise is amplified in the early phase of infection

**Figure 4:**
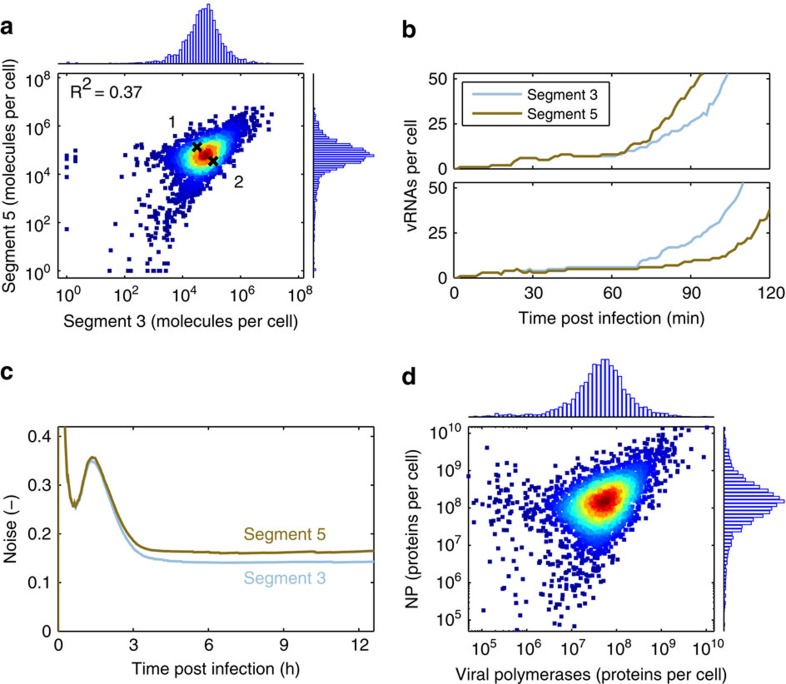
Origin of noise in virus replication. Simulation results for an infection at an MOI of 10 are shown. (**a**) vRNA level of segments 3 and 5 in individual infected cells at 12 h.p.i. Black Xs and numbers correspond to the example cells shown in **b**. Colours from blue to red indicate higher density. Histograms of the data in the *x* and y direction are provided. (**b**) Early dynamics of segment 3 and 5 vRNA for the two cells indicated in **a** (1: upper panel; 2: lower panel). (**c**) Noise in vRNA levels over the course of an infection. The noise was calculated by dividing the s.d. of log_10_ vRNA levels by their mean (see equation (26) for details). (**d**) Number of viral polymerases and NP proteins in individual infected cells at 12 h.p.i. Colours indicate density. Histograms of the data are provided.

As single-cell data confirm the presence of large variations in vRNA levels predicted by our simulations, we next used the model to study the origin of this variability. In agreement with the measurements, we find a positive correlation between the vRNAs of most genome segments in infected cells with the exception of segment 7 (Fig. 4a; Supplementary Fig. 7). In this context, the weaker correlation seen in the model may result from the fact that we did not include cellular factors such as protein content, which represent sources of extrinsic noise, because quantitative information on most of these factors is not available.

According to the model, segment levels start to differ in the early stage of IAV infection about 30–60 min post infection, after parental vRNPs have reached the nucleus and begin to replicate as independent functional units (Fig. 4b). While small in the beginning, these differences grow rapidly over the course of the next hours. In fact, noise is almost exclusively generated in the early phase of infection, when molecule numbers are low and viral transcription and replication take place (Fig. 4c). In particular, the replication of viral genome copies introduces large fluctuations, as it comprises the autocatalytic synthesis of vRNA from cRNA and vice versa. In the model, heterogeneity in vRNA levels also results in large differences in viral mRNAs and protein abundance (Fig. 4d). The viral polymerase and NP protein, for instance, vary by up to three orders of magnitude causing further variability in the cell population. Taken together, these results suggest that infected cells start to differ in their vRNA content during early infection due to the noise intrinsic to biochemical reactions. This noise is amplified by the autocatalytic mechanism of vRNA replication, propagates to viral protein levels and affects virus production.

### Virus release is determined by extrinsic and intrinsic noise

**Figure 5:**
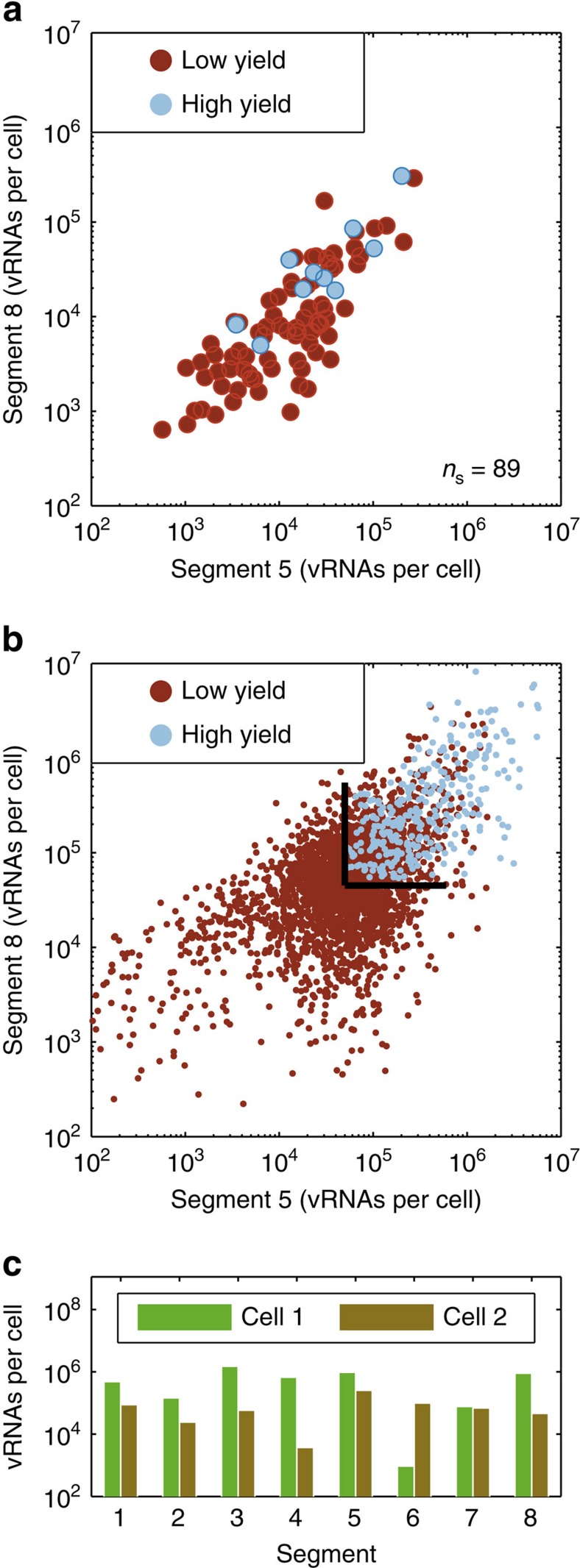
Effect of vRNA level on virus titre. (**a**) Experimental results of the dependency of virus yield on vRNA level. Cells were infected at an MOI of 10 and simultaneously assayed for their virus titres (by the plaque assay) and vRNA levels of segments 5 and 8 (by RT–qPCR) at 12 h.p.i. High-yielding cells (upper 10% of cells with respect to progeny virus release) are indicated in blue and all remaining cells are coloured in red. The illustration includes pooled data of multiple independent experiments (*n*=4). *n*_S_ indicates the number of single-cell measurements. (**b**) Simulated levels of segment 5 and 8 vRNAs in relation to the virus yield. High-yielding cells (upper 10%) are coloured in blue and all remaining cells are shown in red. Black lines indicate the influence of intrinsic noise on virus production. (**c**) RNA levels of two example cells from the simulation in **b** that showed high levels of segments 5 and 8 but were of the low-productive phenotype.

To elucidate the effect of noise on virus release experimentally, we determined the virus yield of an infected cell and, at the same time, its vRNA content. We find that high-yielding cells (upper 10% of cells with respect to PFU) almost exclusively contain elevated vRNA levels with a high intersegment correlation (Fig. 5a). Therefore, we conclude that extrinsic noise, which affects the level of all individual vRNAs in a cell evenly, contributes to differences in virus titres between infected cells.

Surprisingly, some low-productive cells showed high vRNA levels based on the two segments that were measured. We hypothesized that these cells may contain low levels of an unmeasured segment and that this segment ultimately limits their virus yield. This hypothesis was confirmed by our model, in which a similar population of low-productive cells was found even when two genome segments were present at high levels (Fig. 5b). In particular, simulation results of two exemplary cells show a high level of segment 5 and 8, but these cells suffered from a deficiency in segment 4 or 6, respectively (Fig. 5c). As a result, the two cells produced only few infectious virions. Hence, random fluctuations in individual vRNA levels, caused by intrinsic noise, can impair virus titres. This can also be seen in the triangular shape of the high-productive population at the low end of this population (black lines in Fig. 5b). It demonstrates that the virus yield of an infected cell can be limited by the least-abundant vRNA of the two depicted segments. A misbalance between these segments is the result of intrinsic noise in virus replication. Taken together, both extrinsic and intrinsic noise in virus replication affect the virus titre of an IAV-infected cell.

### Single-hit infections are predominantly non-productive

As we could show that virus production is influenced by extrinsic and intrinsic noise at high MOI, we next turned towards low-MOI infections, where stochastic effects should be more pronounced. Such a scenario is, however, hard to address in our experimental system since a low MOI would result in culture plates that mainly contain uninfected cells. Hence, the single-cell assay would be unable to generate a sample size that is large enough to allow us to draw valid conclusions. Therefore, we focused on stochastic simulations to explore low-MOI scenarios in more detail. In particular, we considered cells that were inoculated with a single virus particle (that is, single-hit infections). For the sake of simplicity, we refer to this scenario as an MOI of 1 in the following. However, it may not necessarily correspond to an experimental infection carried out at an MOI of 1, as the MOI in such experiments is typically based on infectious virus titres that were determined by a biological assay. One infectious unit in such an experiment is the particle concentration that leads to productive infection in 63% of cells, which may receive one or more infectious units (based on the Poisson distribution).

**Figure 6:**
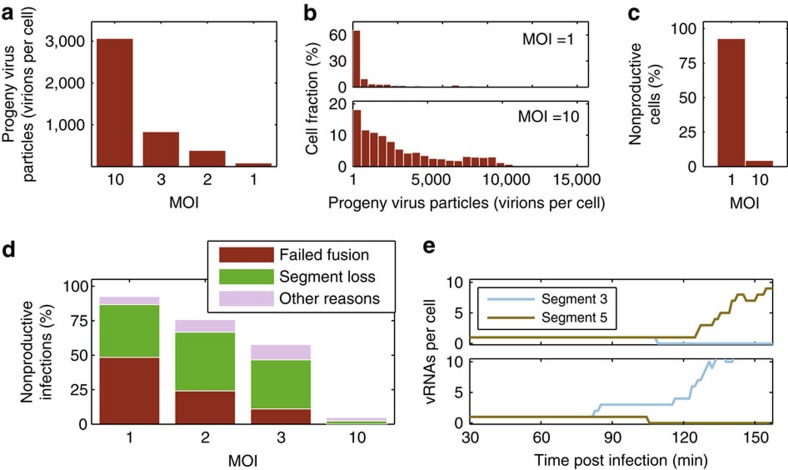
Increase in noise at low MOI. Simulation results at 12 h.p.i. for infections at different MOIs are shown. (**a**) Average number of progeny virions per infected cell after a single round of infection. (**b**) Histogram of the number of progeny virions at an MOI of 1 (upper panel) and 10 (lower panel), respectively. Only productive cells are shown. (**c**) Fraction of non-productive cells at an MOI of 1 and 10. (**d**) Probability that an infected cell does not release infectious virus progeny until 12 h.p.i. for different MOIs. The probabilities that virus fusion fails and that at least one viral genome segment is absent are indicated. (**e**) Early vRNA dynamics in two exemplary non-productive cells (upper and lower panel, respectively) that were infected at an MOI of 1 and in which virus fusion was successful.

Intriguingly, in our simulations, single-hit infection causes the average yield of an infected cell, after a single round of infection, to decrease 35-fold (Fig. 6a). This decrease has two reasons. On one hand, we observe a higher number of low-productive cells, which account for a 2.6-fold reduction in the mean cell-specific virus yield (Fig. 6b). This trend towards lower productivity is mainly caused by a decrease in the number of infecting genome copies, resulting in a slower onset of vRNA synthesis and an increase in noise. On the other hand, there is a significant increase in non-productive infections (Fig. 6c). In particular, ≈93% of the single-hit infections do not result in the release of infectious virus progeny (compared with only 4% at an MOI of 10). In nearly half of these infections, parental virions do not reach the cytoplasm because they fail to fuse with the endosomal membrane (Fig. 6d). This model prediction is based on published experimental data on IAV entry^16^,^17^. Surprisingly, there is a second major reason for non-productive infections, the loss of a genome segment (Fig. 6e; Supplementary Fig. 8). More precisely, the degradation of vRNA in the nucleus (for example, by cellular nucleases) can deprive the cell of an individual genome segment. Such cells are unable to release infectious virus progeny, as the resulting particles would lack a significant part of the viral genome.

**Figure 7:**
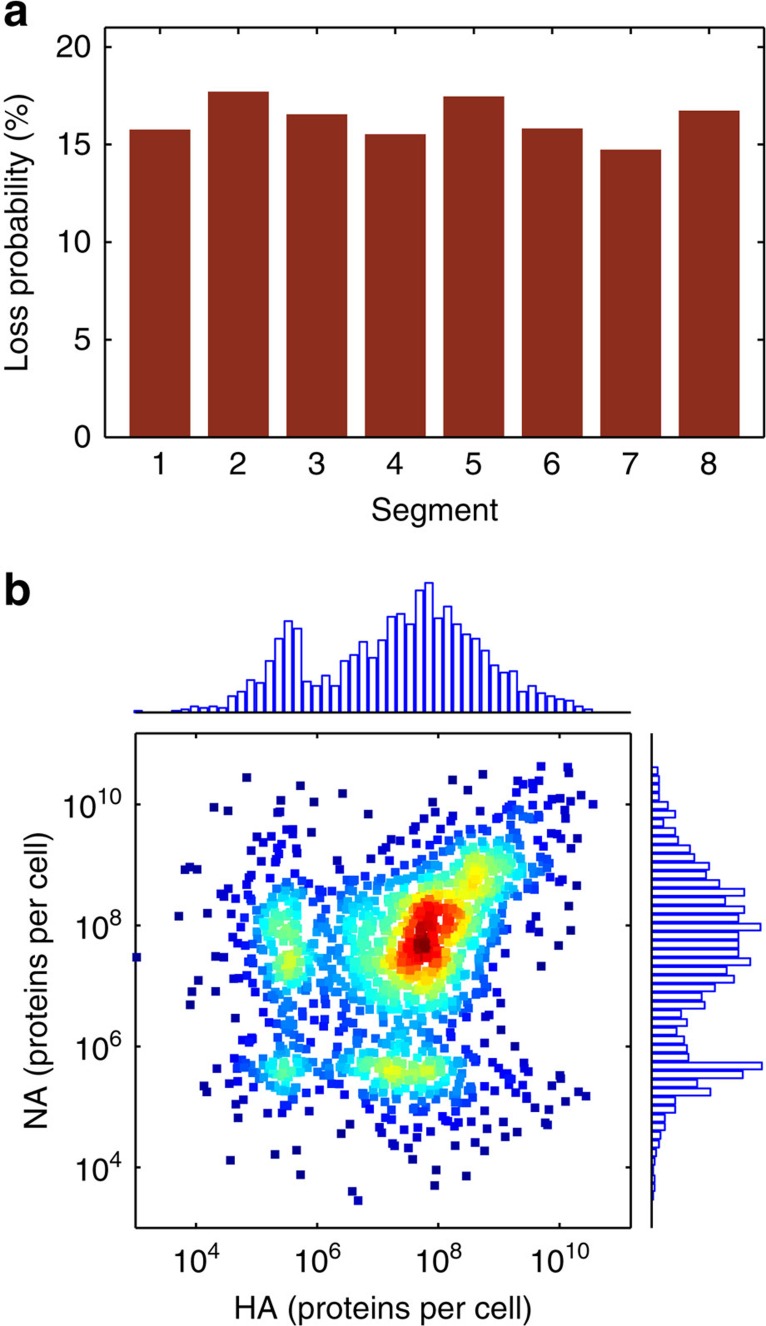
Loss of genome segments in simulations. (**a**) Probability that the indicated segment is absent at 12 h.p.i. in a cell that was infected by one virus particle, which successfully underwent virus fusion. (**b**) Number of NA against HA proteins at 12 h.p.i. for an infection at an MOI of 1. Colours from blue to red indicate higher density. Histograms of the data in the *x* and *y* direction are provided.

The probability that a genome segment is lost depends on the ratio of vRNA synthesis and degradation, which we estimated previously from real-time RT–qPCR data^15^. In our simulations, this probability is between 15 and 18% assuming that exactly one complete set of vRNAs reaches the nucleus (Fig. 7a). However, before an incoming genome segment is degraded, primary transcription from the parental vRNP may occur, such that viral mRNAs are produced. Translation of these few mRNAs can create small amounts of the corresponding protein, resulting in distinct cell populations with respect to viral protein content (Fig. 7b). In particular, in the model we observe a large population of cells that highly expresses haemagglutinin (HA) and neuraminidase (NA), but also cells that show a 200-fold reduction in the level of either protein or of both. The low protein levels are caused by the loss of the corresponding genome segment. In summary, modelling suggests that single-hit infection can lead to a large number of non-productive cells due to virions that do not fuse with endosomes (in ≈48% of the cells) and the loss of genome segments by random RNA degradation (in ≈38% of the cells). This causes additional cell-to-cell heterogeneity with respect to viral protein content and virus production at low MOI, besides the noise originating from vRNA synthesis.

## Discussion

Most experimental assays in virology sample a large cell population to obtain a measurement. By contrast, in this study we investigated the cell-to-cell variability in IAV infection using a combination of single-cell experiments and mathematical modelling. Our results reveal a large heterogeneity in vRNA levels between infected cells as well as substantial differences in the copy number of individual viral genome segments within a cell. We also show that this noise in intracellular RNA levels causes a high variability in virus titres and that titre differences are independent of cell size. Thus, influenza virus infection is highly diverse at the single-cell level with the majority of cells releasing only low amounts of virus progeny while others are highly productive.

The plaque titres of individual infected cells in our experiments cover approximately three orders of magnitude, with yields ranging from 1 to 970 PFU. A similarly high variability in virus yields has been observed in an early study for Western equine encephalomyelitis virus^18^. More recently, single-cell analysis showed that virus titres in VSV infection can span over 300-fold^3^,^4^, and that the production capacity of poliovirus-infected cells varies at least between 269 and 4,225 PFU per cell^8^. In addition, the yield distributions in all these studies are skewed to the left displaying many low-productive cells. This agrees well with our findings for IAV, demonstrating that viral infections are highly heterogeneous and that population averages are an incomplete representation of the system. About 40% of the cells in our experiments release no infectious virus as determined by the plaque assay, although these cells were tested positive for vRNAs. Yet, in simulations only 4% are non-productive at high MOI. This discrepancy seems to be caused by DIPs. At high-MOI conditions, many cells are co-infected by DIPs that completely abolish the release of infectious virions from these cells. Infections with a triple plaque-purified seed virus with no detectable levels of defective interfering RNAs showed ≈1.4% of non-productive cells (Supplementary Fig. 6). However, these infections with a genetically more homogenous and DIP-depleted seed virus still resulted in a large cell-to-cell heterogeneity in virus titres and intracellular vRNA levels.

Our simulations and data indicate that differences in vRNA levels are a major source for the high cell-to-cell variability in virus titres. In particular, the level of the least-abundant genome segment in a cell, which can vary significantly, determines its productivity. By contrast, virus yields in high-MOI infections of poliovirus are largely independent of vRNA content and seem to rather depend on cellular resources^8^. According to our model, a substantial amount of the differences in intracellular vRNA levels arises during the early stage of infection due to the noise inherent to biochemical reactions, that is, due to intrinsic factors. This has also been predicted theoretically for VSV infection by mathematical modelling, which shows that low-abundant species can significantly affect viral replication^7^. Thus, our results support the general notion that viral infections are particularly prone to noise^2^,^3^,^8^. Moreover, the simulations suggest that the autocatalytic mechanism of vRNA replication amplifies the noise generated in the beginning of infection. Similar predictions have been made on theoretical grounds for chemical reactions^19^ and were proposed for phage-infected bacteria^2^. Note that the multiplicative propagation of noise in cascades of catalytic reactions has been shown to result in a log-normal distribution of the involved components^20^,^21^. We find this distribution for most viral genome segments in our experiments, suggesting that the autocatalytic synthesis of vRNA from cRNA and vice versa indeed plays an important role for the variability of RNA levels in IAV replication.

In agreement with the stochastic model, our experiments yield a broad distribution of vRNA levels spanning approximately three orders of magnitude. By contrast, cells infected with poliovirus only show a variation of one to two orders of magnitude^8^, suggesting that influenza viruses are more susceptible to noise. One reason for a larger heterogeneity during the replication of IAVs is their segmented genome. In particular, we observe differences between the vRNA levels of individual genome segments in virus-infected cells in our experiments. Hence, the copy number of the viral genes can vary and is not fixed as during the replication of non-segmented viruses, such as poliovirus or VSV. Together with the observation that high-yielding cells in experiments possess a high level of all measured RNAs, these results indicate that intrinsic noise in genome segment levels can impair virus production.

In our experiments, the levels of individual genome segments inside a cell largely correlate with one another (with the exception of segment 7), suggesting the additional presence of extrinsic noise sources. Surprisingly, however, virus yields are independent of cell size, which contrasts results obtained for VSV infection^3^. The absence of a correlation to cell size also suggests that virus titres may be independent of the cell cycle since both properties are typically linked^22^. Other extrinsic noise sources that could affect virus production are cellular factors required for viral replication. In addition, viral proteins that are essential for RNA replication, such as the polymerase subunits and NP, may cause a positive intersegment correlation, since a change in their level would affect the synthesis of all segments equally.

In contrast to the other vRNAs, the level of segment 7 did not follow a log-normal distribution in our experiments and no correlation to the other vRNAs was apparent. This was also observed in the model (Supplementary Fig. 7) in which a lack of correlation results from the regulatory role of the M1 protein (encoded by segment 7). More precisely, M1 is involved in the nuclear export of vRNPs, which inhibits the activity of the viral polymerase^15^,^23^. This may cause a negative correlation to the other vRNAs, that is, an increase in segment 7 results in an increase in M1, which reduces the synthesis of all cRNAs and, thus, impairs the overall vRNA production. This negative correlation is superimposed onto the positive correlation typically seen between the segments. Experimental prove of this hypothesis is the subject of ongoing studies.

Interestingly, our simulations of single-hit infections yield a large number of non-productive cells. This prediction is in good agreement with experiments at low MOI in which nearly 90% of the cells are incapable of producing infectious virus progeny^24^. In our model, a failure to release progeny virions is, on the one hand, caused by an abortion of virus entry due to virions that do not accomplish fusion in late endosomes. This prediction is based on two experimental studies showing that only ≈50% of the incoming virions successfully undergo fusion^16^,^17^. On the other hand, there is a surprisingly large number of cells in which random RNA degradation results in the loss of a genome segment. Similar observations have been made previously in a stochastic model of a generic virus, where infection failed due to the degradation of essential viral components^25^, and in experimental single-hit infections with vaccinia virus that stopped at various stages of the viral life cycle^26^. Our model predicts that, once in the nucleus, each IAV vRNP has a chance of 84% to replicate successfully. Considering a cell in which only one viral genome set reaches the nucleus, the probability to replicate all eight vRNPs, which is required to release infectious viruses, is thus only 0.84^8^ ≈25%.

In simulations, the early loss of a genome segment causes a 200-fold reduction in the corresponding viral protein(s) to the level generated by primary transcription. This result is particularly interesting in the context of a recent study by Brooke *et al.*, who measured the expression of four viral proteins during low-MOI infection with IAV. The authors found that in most infected cells the level of at least one of these proteins was below the detection limit of flow cytometry^24^, that is, most cells failed to express all four proteins. On the basis of these findings, Brooke *et al.* hypothesized that IAVs may exist as a population of semi-infectious virions with protein expression during single-hit infections being affected by internal deletions in genome segments (for example, defective interfering RNAs), non-sense or lethal mutations, or the lack of a vRNA in virus particles. However, our simulations put forward an alternative explanation. In particular, the loss of genome segments at low MOI due to random RNA degradation may contribute substantially to the observed failure to express viral proteins. More precisely, our model predicts a probability of 84 and 25% for the successful amplification of a specific genome segment and all eight vRNPs, respectively. This is comparable to the results of Brooke *et al.*, who find that the average expression frequency of a particular protein in an infected cell is 78.1% and the chance to express the products of all eight genome segments 13.8% (ref. 24). In this context, the slight overestimation by our models (the deviation between 84 and 78.1%) may very well result from defects in vRNAs or virus particles, as outlined above. Our estimation would, hence, represent an upper limit for successful protein expression and virion release at low MOI, since our model assumes that all infecting viruses contain a full set of functional vRNPs. This also implies that even if all parental viruses comprise a complete genome set, many cells would not produce infectious virus progeny during low-MOI infection due to stochastic effects. However, during subsequent rounds of infection, these cells could be infected a second time causing them to contribute to total virus titres.

In summary, we have developed an experimental approach to analyse the infection of individual cells with IAV, which reveals significant heterogeneity in viral replication that is driven by extrinsic as well as intrinsic factors. Furthermore, we have derived a novel stochastic mathematical model for the intracellular viral life cycle that not only reproduces these measurements but also suggests that infections at low MOI feature a substantial proportion of non-productive cells. The outcome of such infections may, hence, be a highly stochastic process challenging current beliefs that mean field measurements and simulations (that is, approaches that characterize cell populations) are an accurate representation of low-MOI infections. Studying the cell-to-cell variability in IAV replication in more detail may reveal how the virus establishes itself in a host and whether it has adopted mechanisms to counteract noise.

## Methods

### Cells and viruses

Adherent MDCK cells (ECACC, #84121903) were maintained in Glasgow minimum essential medium supplemented with 10% fetal calf serum and 1% peptone at 37 °C in a 5% CO_2_ atmosphere. Serum-free infection medium comprised of Glasgow minimum essential medium, 1% peptone and porcine trypsin at a concentration of 5 BAEE U ml^−1^ was used for infection experiments. Influenza virus strain A/Puerto Rico/8/34 was supplied by the National Institute for Biological Standards and Control (code: 06/114). The titre of the seed virus utilized was determined by the 50% tissue-culture-infective dose (TCID_50_) assay on MDCK cells. MOIs were calculated based on the TCID_50_ titre.

### Isolation of infected single cells

Confluent cells in 35-mm dishes were washed twice with PBS and infected at an MOI of 10 in 250 μl of infection media for 1 h. During incubation, the dishes were rocked gently every 20 min to keep the monolayer moist and to distribute viruses evenly. The infection medium was then increased to 2 ml followed by incubation for an additional 1.5 h. The inoculum was removed and cells were washed twice, followed by addition of 500 μl of a trypsin solution (0.05% trypsin and 0.02% EDTA in PBS). Trypsinization was performed for 13 min and stopped by adding 500 μl of cell-maintenance media (containing 10% fetal calf serum). The homogenized cell suspension was serially diluted in pre-warmed infection media (37 °C) to obtain a cell concentration of one cell per 50 μl. Subsequently, 50 μl of this dilution were added to each well of a 384-well plate (pre-warmed to 37 °C). At 12 h.p.i., the plates were briefly centrifuged at 300*g* to ensure that cells are located at the bottom of the plate. Wells containing single cells were then identified by phase-contrast microscopy.

### Investigation of virus yield and cell size

To determine virus yield and cell size both at the same time, we isolated single infected cells in non-binding 384-well plates (Greiner, #781901). The usage of this type of plate decreases a loss in virus titres, which can be caused by unspecific adsorption of virions to the plastic surface of a well. It also suppresses adherence of cells and causes them to remain spherical. Thus, cell diameters could be measured using microscopic images and the software Axiovision V 1.1 (Zeiss). Thereafter, the supernatant was immediately subjected to plaque assay analysis to quantify virus titres.

### Investigation of intracellular vRNA levels and virus yield

Single infected cells were isolated in tissue-culture-treated 384-well plates (Greiner, #781182) to investigate intracellular vRNAs. This type of plate enables cell adherence, which is necessary for washing and subsequent cell lysis. After incubation, adherent single infected cells were washed twice with PBS. Five microlitre of lysis buffer (Thermo Scientific, #B14) diluted to 1 mg ml^−1^ bovine serum albumin (BSA) in nuclease-free water was then added to the cells. The plate was sealed and immediately stored at −80 °C until real-time RT–PCR. Lysis of cells in BSA solutions by freeze–thawing results in efficient cell disruption, high RNA stability and enhanced RT efficiency^27^.

To investigate virus titre and intracellular vRNA content of an individual cell, we isolated single infected cells in non-binding 384-well plates. The occurrence of adherent single cells in this type of plate is rare; however, it facilitates washing and subsequent cell lysis. Moreover, it decreases the loss in virus titres caused by unspecific adsorption of virions to the plastic surface of a well. After incubation, the supernatant was immediately applied to plaque assay. Cells were then washed twice, followed by addition of 5 μl of lysis buffer. The plate was sealed and straightaway stored at −80 °C until real-time RT–PCR.

### Plaque assay

Whole volumes of single-cell samples were subjected to plaque assay analysis. Specifically, we investigated two dilutions, containing 90 and 10% of the total sample in 250 μl of infection media. For population-based experiments, we prepared serial 10-fold dilutions of the samples. Two hundred and fifty microlitres of each dilution were then incubated on confluent MDCK cells in six-well plates. During 1 h of incubation, the plates were rocked gently every 20 min. The inoculum was removed and cells were overlaid with infection media containing 1% agar (pre-warmed to 45 °C). Incubation took place for 4 days. Subsequent to methanol fixation and removal of the overlay, cells were stained with a 0.2% crystal violet solution. The virus load of the sample, expressed as PFU, was determined using an inverted microscope. Owing to a small volume and a low quantity of virus of the single-cell samples, we performed the plaque assay in single measurements.

### Real-time RT–qPCR

For quantification of intracellular genomic vRNA, we utilized real-time RT–qPCR and a primer combination enabling polarity- and gene-specific amplification of individual vRNAs^28^. Primers for RT and qPCR are listed in Supplementary Tables 1 and 2, respectively. To facilitate an absolute quantification, we generated RNA reference standards and calculated the number of vRNAs per cell based on calibration curves. Detailed procedures of the synthesis of RNA reference standards, as well as the calculations for absolute quantification are described in the Supplementary Methods.

For RT, we combined 1 μl of a lysed single cell with 0.5 μl of a 10-mM dNTP solution and 0.5 μl of a 10-μM RT primer, and increased the volume to 6.5 μl with nuclease-free water. The mixture was incubated at 65 °C for 5 min and subsequently cooled down to 55 °C for 5 min. 1 × RT buffer (comprising 50 mM Tris-HCl (pH 8.3), 75 mM KCl, 3 mM MgCl_2_ and 10 mM dithiothreitol) and 50 U Maxima H Minus Reverse Transcriptase were then added to a final volume of 10 μl (all reagents from Thermo Scientific). RT was performed at 60 °C for 30 min and terminated at 85 °C for 5 min. Serially 10-fold-diluted RNA reference standards (1-1 × 10^−7^ ng), containing 350 fg cellular total RNA, were reverse transcribed in parallel. cDNA products were then diluted to 20 μl in nuclease-free water before qPCR analysis.

The Rotor-Gene Q real-time PCR cycler (Qiagen) was utilized for qPCR measurements. The reaction mixture (10 μl) contained 1 × Rotor-Gene SYBR Green PCR Kit (Qiagen), 500 nM of each primer (Thermo Scientific) and 3 μl of diluted cDNA. The temperature profile included denaturation at 95 °C for 5 min, followed by 40 cycles of amplification in two steps at 95 °C for 10 s and 62 °C for 20 s. Primer dimer signals did not appear within 40 cycles of amplification (analysed by melting-curve analysis). QPCR efficiencies were between 90-100%. Measurements with *c*_T_ values greater than the values obtained from the corresponding standard curve were excluded from further analysis. Fractions of these non-quantifiable data points and of measurements with no *c*_T_ value are listed in Supplementary Table 3.

### Population-derived measurements

Confluent cells in 35 mm dishes were washed twice with PBS and infected at an MOI of 10 in 250 μl of infection media. After 1 h of incubation (gentle rocking every 20 min), the supernatant was removed and cells were washed twice. Two millilitres of infection media were added and cells were incubated at 37 °C. At 12 h.p.i., the supernatant was immediately subjected to plaque assay analysis. Cells were then washed twice with PBS. Lysis of cells and RNA extraction were performed using the INSTANT Virus RNA Kit (Analytik Jena) according to the manufacturers' instructions. RNA samples were then investigated by real-time RT–qPCR. Cell-specific virus titres and vRNA levels were calculated based on the viable cell count at the time point of infection.

### Mathematical model

Our stochastic model of IAV replication is based on a deterministic description of the system developed previously by our group^15^. For a detailed discussion of the equation system and its agreement with experimental data, the reader is referred to the original publication. We extended this model by accounting explicitly for the eight genome segments and introduced minor modification to reduce computational costs. However, none of these changes significantly affects simulation results in a deterministic setting (Supplementary Fig. 9). The overall structure of the resulting model is summarized in Fig. 2a. In the following, we provide a complete list of its equations.

First, virus particles in the extracellular medium (*V*^Ex^) bind to free binding sites (i.e. sialic acid residues) on the cell surface (*B*_n_).


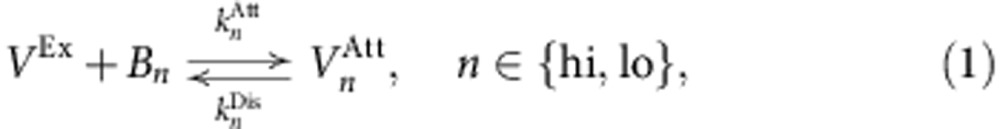



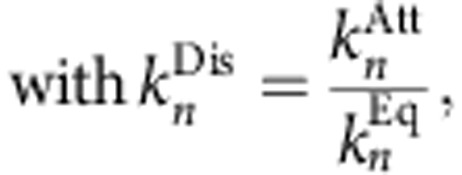


where 
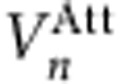
 denotes the attached virus particles. On the basis of experimental data^29^, we distinguished two types of binding sites: high-affinity (*n*=hi) and low-affinity (*n*=lo) sites. The virus can bind to these sites with rate 
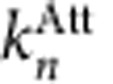
 or dissociate from them with rate 
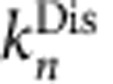
. The latter rate follows from the equilibrium constant (
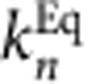
). After binding to the cell, virus particles can enter by receptor-mediated endocytosis.


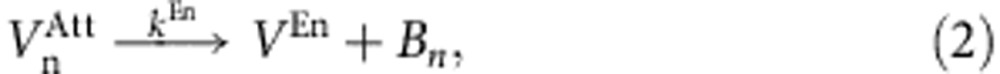


where *V*^En^ denotes virions in endosomes and 
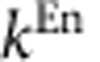
 is the endocytosis rate. As described previously, we assumed a fast recycling of receptors such that binding sites become available when virions undergo endocytosis^15^. Once inside the cell, virus particles can either fuse with the endosomal membrane or are degraded in lysosomes if they are fusion incompetent.


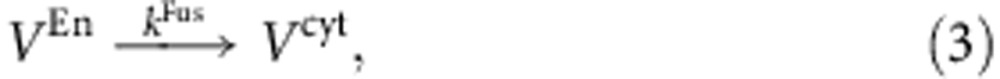



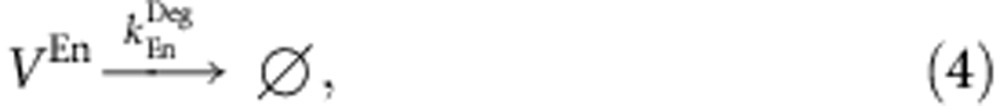



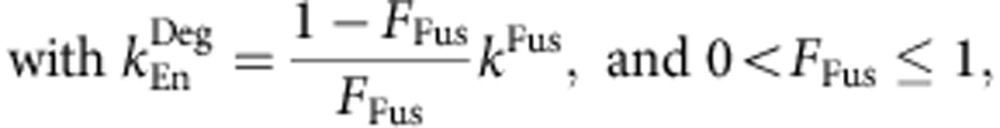


where 
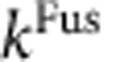
and 
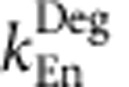
 are the rates of fusion and degradation, respectively, and *V*^cyt^ denotes a cytoplasmic complex that comprises the eight parental vRNPs released upon fusion. The latter variable was implemented due to recent experiments, which show that parental genome segments travel together through the cytoplasm until they reach the nucleus^30^. Also note that we introduced the fraction of fusion-incompetent viruses (*F*_Fus_) based on R18-labelling experiments in which half of the infecting virions fail to reach the cytoplasm^16^,^17^. Following fusion, parental vRNPs can enter the nucleus where they act as independent replication units (
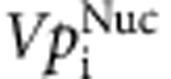
), each containing one of the eight genome segments *i*.


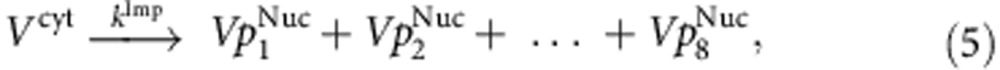


where *k*^imp^ denotes the nuclear import rate.

Inside the nucleus, parental vRNPs start to transcribe viral mRNA (
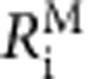
) and, according to the stabilization hypothesis^15^,^31^, also cRNA (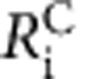).


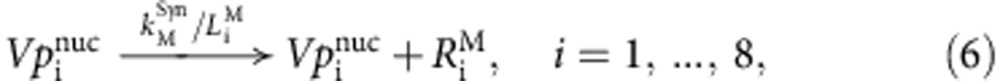



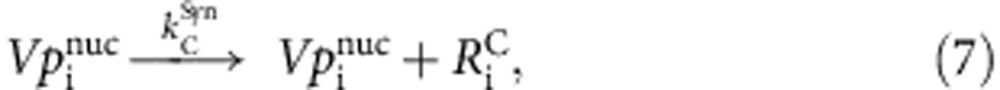


where 
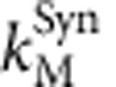
 and 
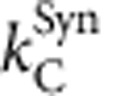
 are the synthesis rates of mRNA and cRNA, respectively. Since the number of influenza virus mRNAs is negatively correlated with their length, that is, shorter mRNAs are more abundant^32^, we scaled the transcription rate with mRNA length (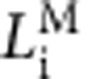). After their synthesis, viral mRNAs enter the cytoplasm (a process not modelled explicitly because mRNA export is fast^33^). There, they are translated into viral proteins. The mRNAs of segment 1−3 encode for the three subunits of the vRNA-dependent RNA polymerase (*P*_RdRp_). To ease the computational burden, we lumped subunit synthesis and polymerase assembly into a single reaction.


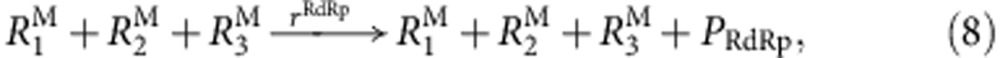


with


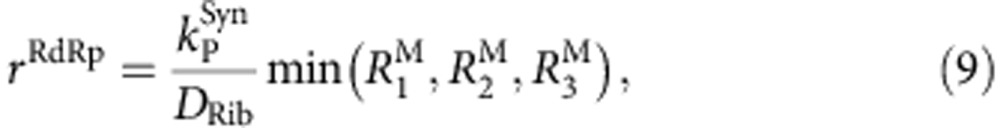


where 
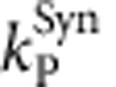
 denotes the protein synthesis rate. Since multiple ribosomes can translate a single mRNA, we introduced the distance between two adjacent ribosomes on an mRNA (*D*_Rib_). The protein synthesis rate is proportional to the speed with which the ribosomes cover this distance. Note that using equations (8) and (9) implies that polymerase formation is limited by the synthesis of the polymerase subunits and that this synthesis is proportional to the least abundant of the three mRNAs encoding the subunits. The mRNAs of segment 4−6 encode for the HA (*P*_HA_), NP (*P*_NP_) and NA (*P*_NA_) proteins, respectively.


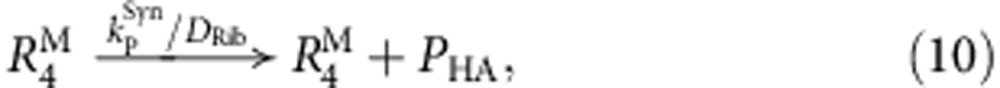



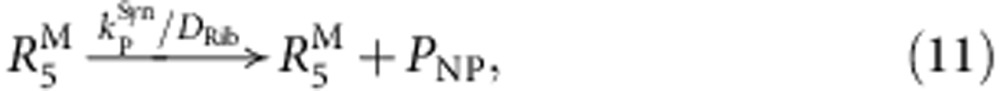



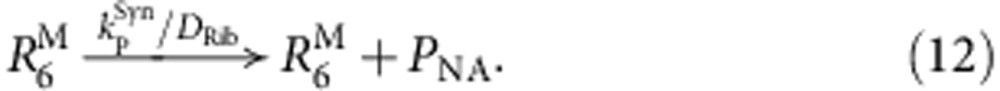


Their synthesis occurs as described above. The M1 (*P*_M1_), M2 (*P*_M2_) and NEP (*P*_NEP_) proteins are derived from spliced mRNAs of segments 7 and 8.


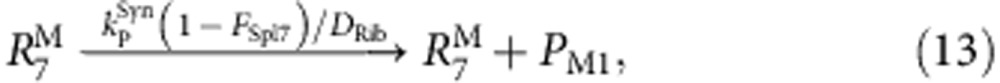



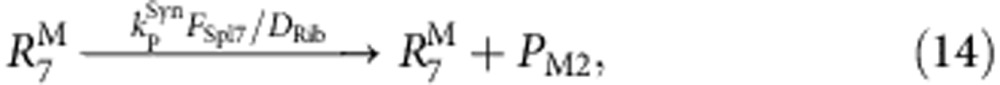



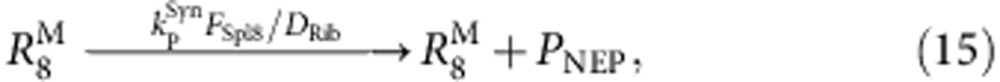


where *F*_Sp17_ denotes the fraction of mRNAs of segment 7 that encodes for M1. Similarly, *F*_Sp18_ is the fraction of NEP-encoding mRNAs of segment 8. Note that we omitted the synthesis of nonstructural proteins to reduce computational costs.

Following their synthesis, viral polymerases and NP proteins can enter the nucleus, where they encapsidate nascent cRNA.


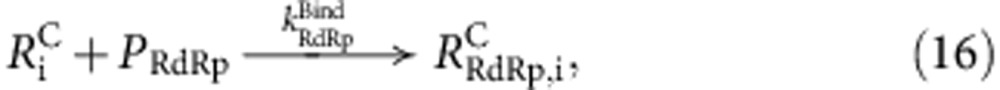



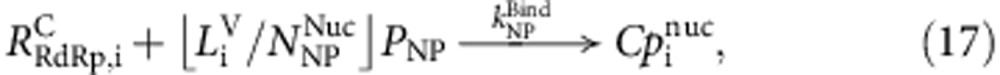


where 
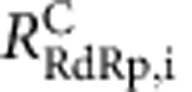
 and *Cp*_i_ are RdRp–cRNA complexes and cRNPs, respectively, which contain the cRNA of segment *i*. Here, each cRNA binds one tripartite polymerase complex with rate 
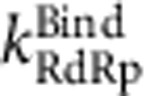
 and multiple NP proteins with rate 
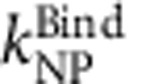
. The number of NP molecules in one cRNP follows from the length of the segment (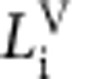) and the number of nucleotides bound by one NP protein (
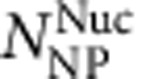
). We rounded this number down to the next smaller integer, since stochastic models only account for discrete state variables. In the second step of genome replication, cRNPs synthesize new vRNAs (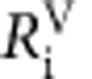), which are again encapsidated by viral proteins to form progeny vRNPs (
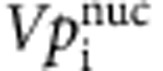
).


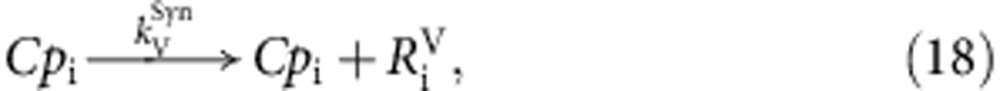



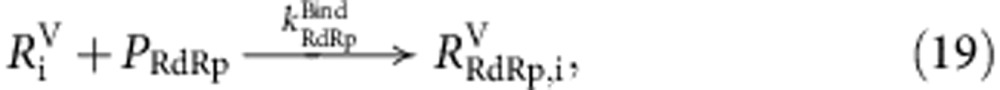



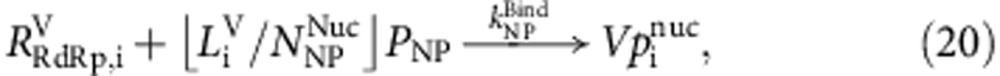


where 
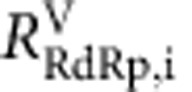
 denotes an RdRp–vRNA complex and 
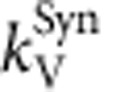
 is the vRNA synthesis rate. The newly produced vRNPs can either participate in mRNA and cRNA synthesis (equations (6) and (7)) or bind to M1 proteins to form M1–vRNP complexes (
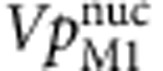
).


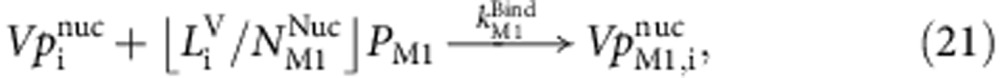


where 
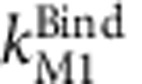
 denotes the M1-binding rate. The number of proteins required to cover the vRNP is calculated by the length of each genome segment (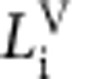) and the number of nucleotides bound by one M1 molecule (
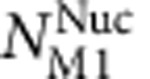
). Note that M1 binding is assumed to inactivate vRNPs in preparation of nuclear export such that M1–vRNP complexes do not participate in mRNA and cRNA synthesis^15^. Subsequently, the NEP protein binds to the inactivated vRNPs and facilitates their transport to the cytoplasm.


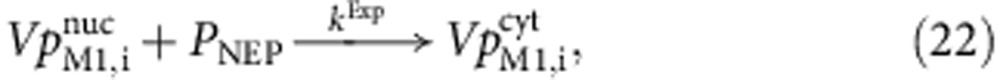


where 
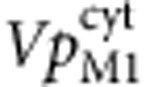
 denotes the NEP–M1–vRNP complex in the cytoplasm and *k*^Exp^ is the export rate. Since NEP is not required in stoichiometric quantities^34^, we assumed that one molecule is sufficient to induce export. Also, the actual transport process was assumed to occur fast such that NEP binding is the rate-limiting step.

Inside the cytoplasm, newly produced vRNPs travel to the plasma membrane, where they meet with the viral proteins to produce progeny virus particles (*V*^Rel^). As the molecular details of this process remain elusive, we modelled particle assembly as a single reaction step that requires the eight vRNPs and all structural viral proteins.


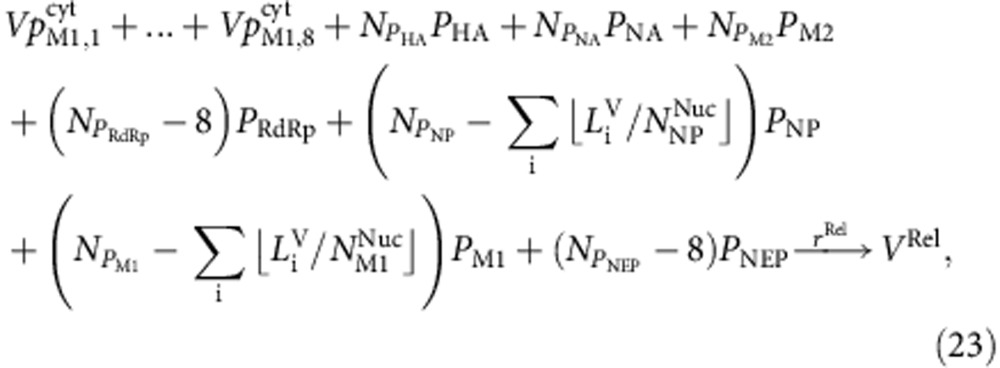


where 
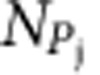
 with 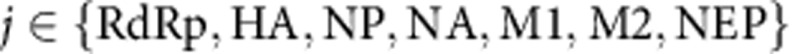 denotes the number of viral proteins inside a virus particle. As some of these proteins are also present in vRNPs, the number of proteins required for packaging was calculated by subtracting the amount of proteins in a complete set of eight vRNPs from the total protein number per particle. In analogy to the original model^15^, the release rate (*r*^Rel^) is given by the following equation.


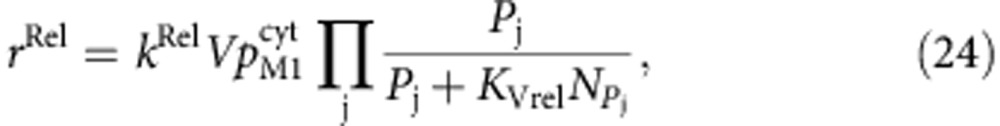



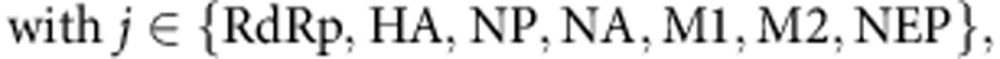


where *k*^Rel^ is the specific virus release rate and *K*_Vrel_ denotes the number of virus particles for which viral components must be present to reach half the maximum release rate. The influence of viral proteins is considered by Michaelis–Menten-like terms. In addition to the reactions outlined above, all components containing vRNA (with the exception of *V*^cyt^) are subject to degradation.


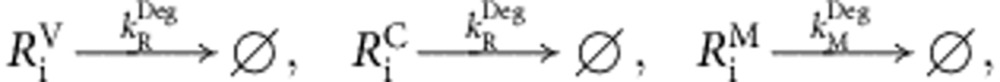



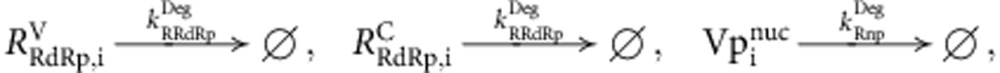



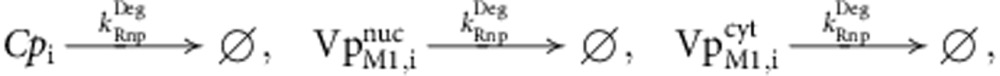


where 
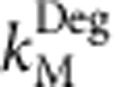
 and 
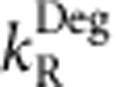
 are the degradation rates of mRNA and nascent cRNA and vRNA, respectively. 
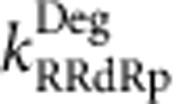
 denotes the degradation rate of cRNA and vRNA complexes with the viral polymerase and 
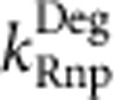
 is the degradation rate of RNPs. Note that, according to the cRNA-stabilization hypothesis, encapsidation by viral proteins protects nascent cRNA (and perhaps also vRNA) from nuclease digestion 
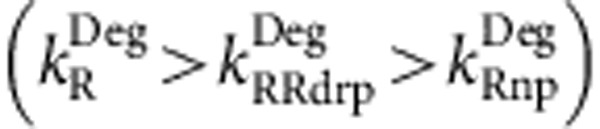
. The decay of incoming vRNPs (*V*^cyt^) was omitted based on the constant vRNA level observed in cells treated with the protein synthesis inhibitor cycloheximide^31^. Furthermore, protein degradation was not included as the original model only accounted for the net production of viral proteins^15^.

To determine the total number of vRNAs of segment *i* (
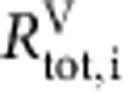
), we summed overall vRNA-containing complexes in an infected cell.


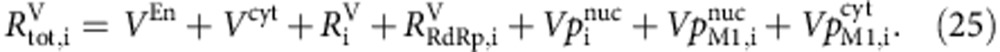


### Computation

We simulated the stochastic model as a discrete jump Markov processes using the stochastic simulation algorithm, also known as the Gillespie algorithm (reviewed elsewhere^35^) and assumed that lumped reactions can also be modelled as a Markov process. Since computational performance is of critical importance, we implemented an improved version of the algorithm, the sorting direct method^36^. In addition, an approximation of the stochastic simulation algorithm, the *τ*-leaping method with efficient step size selection^37^, was used.

For all stochastic simulations, we assumed that a reaction can only occur if all its substrates are available, for example, virus assembly can only take place if a complete set of viral proteins to build at least one virus particle is present. Furthermore, we chose the reaction rates according to mass action kinetics with the exception of viral polymerase production and virus release whose rates are provided in (equations (9) and (24)), respectively. Also note that for reactions where multiple proteins bind in a single step (equations (17), (20) and (21)), first-order mass action kinetics were used and higher-order exponents were neglected.

To obtain a representative sample of the system dynamics, we performed 3,000 simulation runs for each condition. A higher number of runs does not result in a significant change of mean simulation values (Supplementary Fig. 10). The initial number of extracellular virions (*V*^Ex^(*t*=0)) in these runs was set to the MOI indicated in each figure. The parameter values were taken from the original model^15^ and can be found in Supplementary Table 4. For some simulation runs, the loss of segment 7 (encoding M1 and M2) or a low number of M1 proteins prevented an efficient negative regulation of RNA synthesis. This led to an exponential increase in RNA levels and computation time. We, thus, stopped simulations after 5 × 10^10^ iterations at the latest and disregarded such runs for all further analysis, except when calculating the probability of unsuccessful infections and segment loss (Figs 6d and 7a). All model files were handled in MatLab (version 8.0.0.783 R2012b) on a Linux-based system and simulations were performed via the parallel computing toolbox on a Linux cluster.

### Code availability

The entire computer code is available upon request.

### Quantification of noise

To quantify the noise in vRNA levels, we used the coefficient of variation.


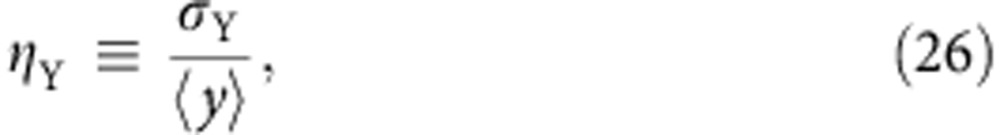


where *η*_Y_ denotes the noise in quantity *Y* with s.d. *σ*_Y_ and mean 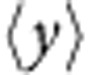. Since the vRNA levels in stochastic simulations follow a log-normal distribution, we defined *Y* as the decadic logarithm of the RNA abundance.

## Additional information

**How to cite this article:** Heldt, F. S. *et al.* Single-cell analysis and stochastic modelling unveil large cell-to-cell variability in influenza A virus infection. *Nat. Commun.* 6:8938 doi: 10.1038/ncomms9938 (2015).

## Supplementary Material

Supplementary Information Supplementary Figures 1-11, Supplementary Tables 1-5, Supplementary Methods and Supplementary References
