# Supplementary material for: Single-cell analysis and stochastic modelling unveil large cell-to-cell variability in influenza A virus infection
Source: Nat Commun. 2015 Nov 20;6:8938. doi: 10.1038/ncomms9938 (PMC4673863; doi:10.1038/ncomms9938)
Supplement: Supplementary Information — Supplementary Figures 1-11, Supplementary Tables 1-5, Supplementary Methods and Supplementary References [file ncomms9938-s1.pdf]

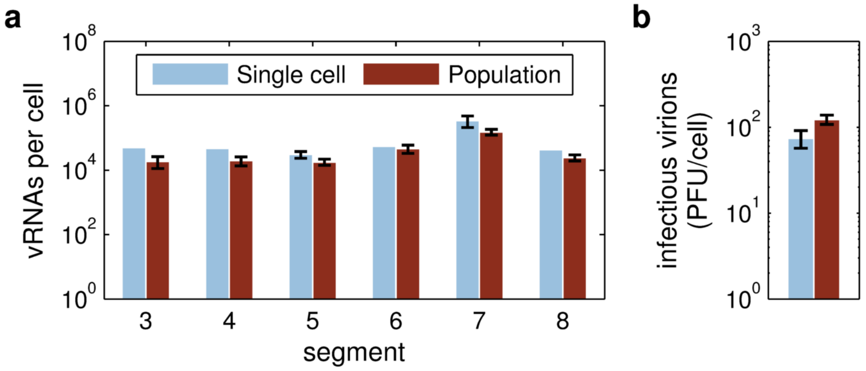

Supplementary Figure 1 | Comparison of single cell- and population-based experiments. Means of single cell- and population-derived measurements of cell-specific vRNA levels for segment 3 to 8 (**a**) and of virus titers (**b**). Cells were infected at an MOI of 10 and analyzed at 12 hpi for their virus yield by plaque assay and intracellular vRNA content by real-time RT-qPCR. Illustrations include data of multiple independent single-cell experiments (n=2 for segment 3, 4 and 8; n=5 for segment 5; n=1 for segment 6; n=3 for segment 7; n=8 for virus yield). Three independent experiments were performed for population-derived measurements. Error bars indicate standard deviations.

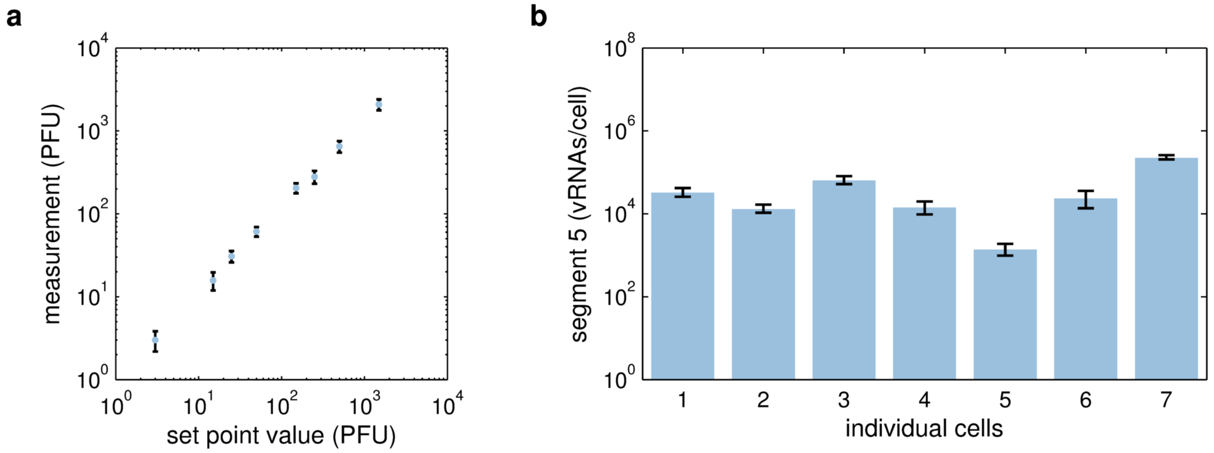

Supplementary Figure 2 | Technical measurement error. Errors were determined by multiple measurements of the same sample in technical replicate (n=4). Error bars indicate standard deviation. (**a**) Measurement error of the plaque assay. Samples derived from diluted seed virus were adjusted to different amounts of PFU (set point value) per test volume of a single cell (50 µL) and subjected to plaque assay. RSDs ranged from 13% to 27%, with a mean RSD of 18%. (**b**) Measurement error of real-time RT-qPCR. Single cells infected at an MOI of 10 were analyzed for intracellular vRNA of segment 5 at 12 hpi by real-time RT-qPCR. RSDs ranged from 12% to 45%, with a mean RSD of 27%.

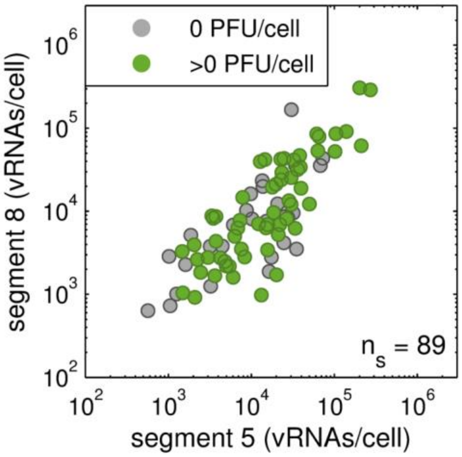

Supplementary Figure 3 | vRNA content of cells with non-detectable virus titer. Single cells infected at an MOI of 10 were simultaneously investigated for their virus yield by plaque assay and for their vRNA levels (of segment 5 and 8) by RT-qPCR at 12 hpi. Cells showing no virus release (0 PFU) are indicated in gray (28 cells) and all remaining cells are colored in green (61 cells). The illustration includes pooled data of multiple independent experiments (n=4). n_S indicates the number of single cell measurements.

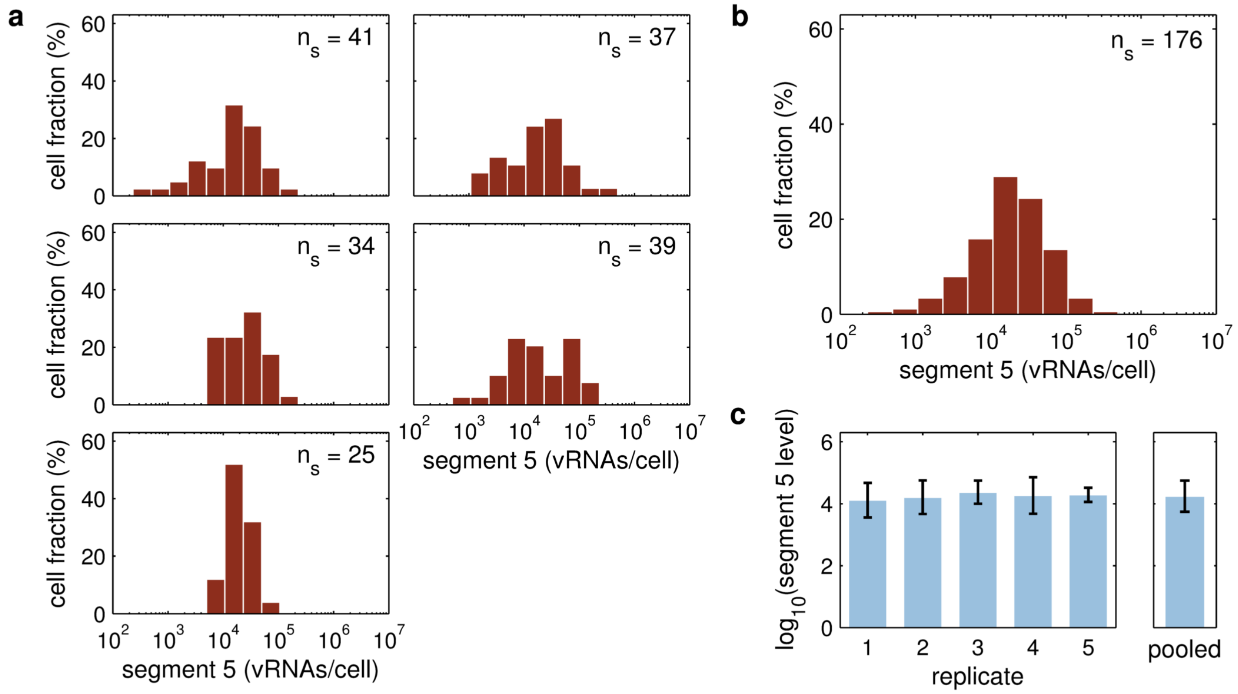

Supplementary Figure 4 | Histograms of segment 5 vRNA level obtained from independent experiments. Single cells infected at an MOI of 10 were analyzed for their vRNA content of segment 5 at 12 hpi via real-time RT-qPCR. n_S indicates the number of cells analyzed. (**a**) Frequency distributions of vRNA levels for five independent experiments. (**b**) Histogram of the vRNA level for data obtained by pooling the independent experiments shown in (a). (**c**) Means and standard deviations of vRNA levels from the independent replicates shown in (a) and the pooled data in (b).

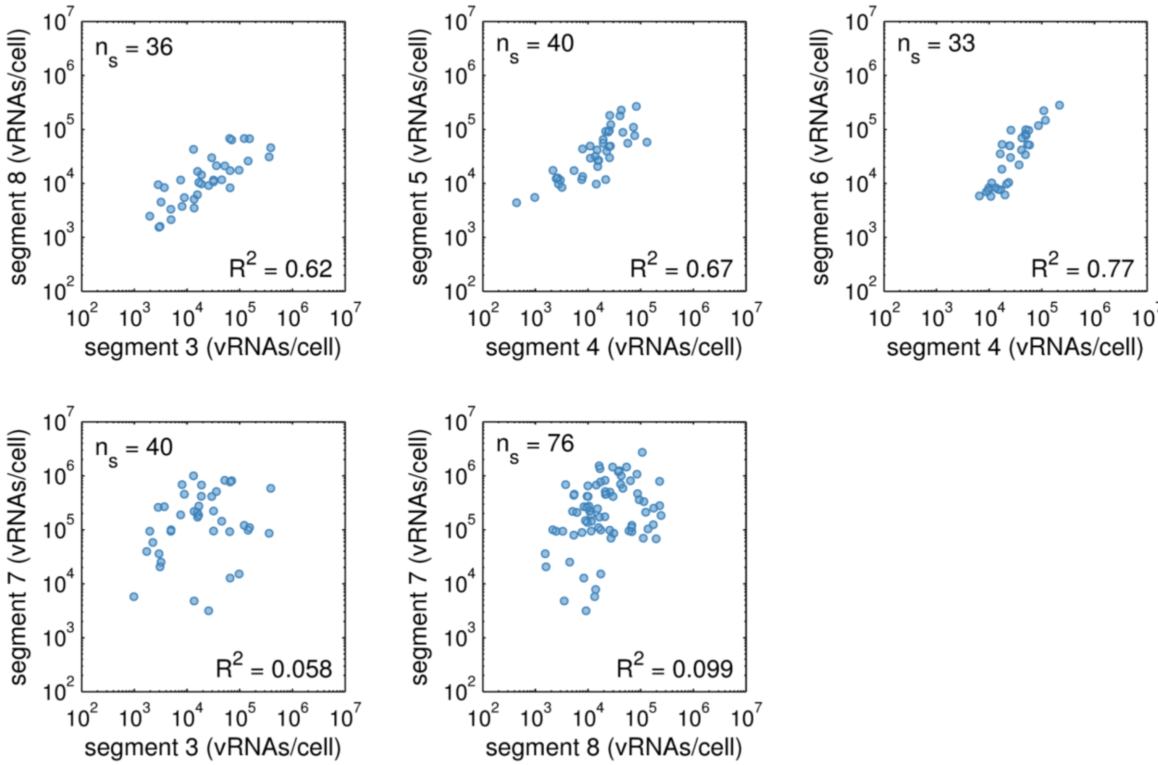

Supplementary Figure 5 | Correlation of vRNA segments. Single cells infected at an MOI of 10 were analyzed for their intracellular vRNA content at 12 hpi by real-time RT-qPCR. The illustrations include pooled data of two independent experiments (scatter plot of segment 7 vs. segment 8) and data obtained from single experiments (remaining scatter plots). The coefficient of determination (R^2) is provided. n_S indicates the number of single cells analyzed.

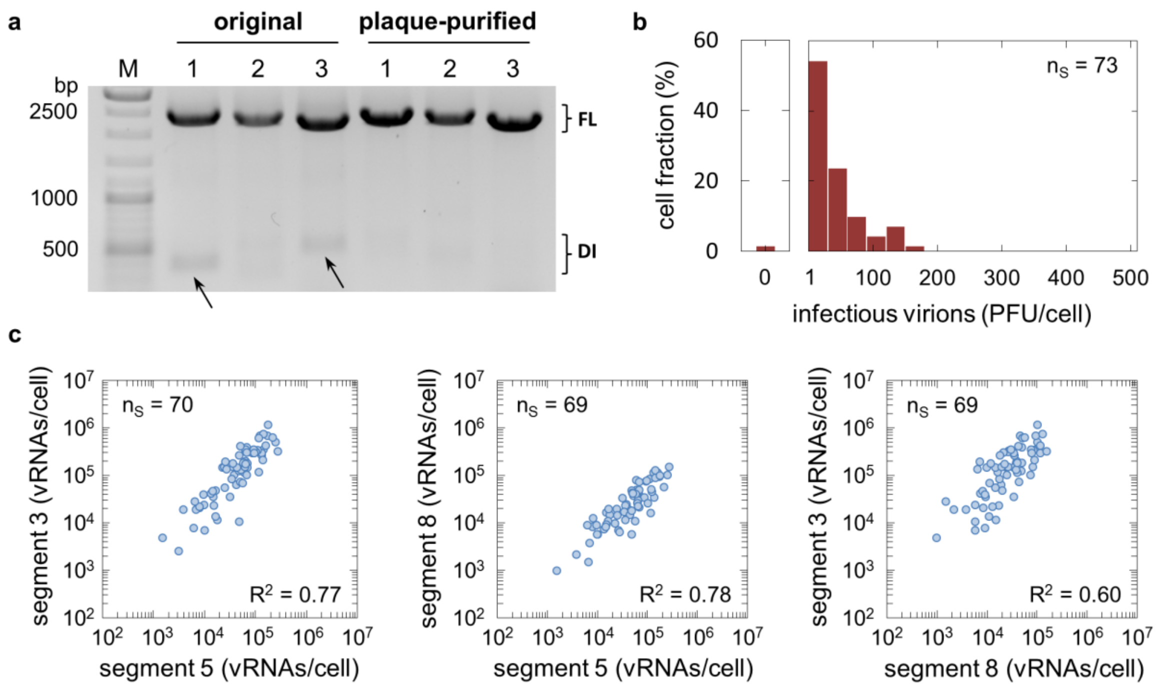

Supplementary Figure 6 | Virus replication in single cells infected with a plaque-purified virus. To reduce the genetic heterogeneity, the original seed virus material used in this work was plaque-purified three times. The purified virus was afterwards expanded in MDCK cells. (**a**) Viral full-length (FL) and defective interfering (DI) RNAs in the original and plaque-purified virus seed. A segment-specific RT-PCR[1] was carried out to amplify the genome segment 1, 2 and 3, from which influenza virus DI RNAs are usually derived from. Numbers represent genome segments 1, 2 and 3 and M indicates DNA marker position. Selected marker bands with corresponding lengths in base pairs (bp) are indicated. Arrows indicate faint bands of DI RNAs. (**b**) Distribution of virus yield. Single cells were infected with the triple plaque-purified virus shown in (a) at an MOI of 10. At 12 hpi, cells were analyzed for their virus titers in the supernatant by using the plaque assay and intracellular vRNA content by real-time RT-qPCR. The first bar on the left of the histogram indicates the fraction of cells that show no virus release (0 PFU). (**c**) Intersegment dependencies of vRNAs. The coefficient of determination (R^2) is provided. n_S indicates the number of single cells analyzed.

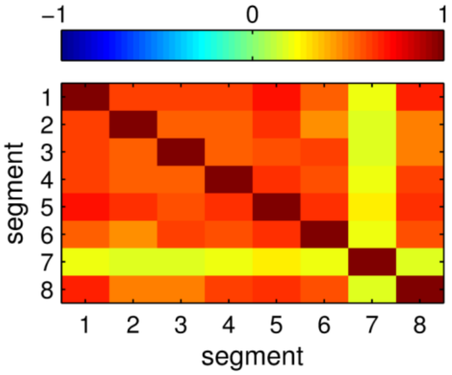

Supplementary Figure 7 | Correlation between different genome segments. Pearson's linear correlation coefficient (R) between the simulated vRNA levels of different genome segments at 12 hpi is shown for an infection at an MOI of 10.

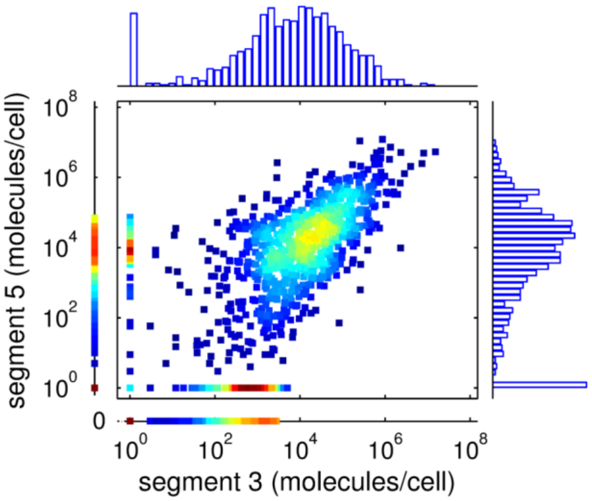

Supplementary Figure 8 | Genome segment levels at low MOI. The simulated vRNA levels of segment 3 and 5 at 12 hpi for an infection at an MOI of 1 are shown. Separate axes on the left and below the scatter plot indicate cells in which segment 3 (≈16% of cells) or 5 (≈17% of cells) is missing, respectively. Values on these axes are not included in the histograms.

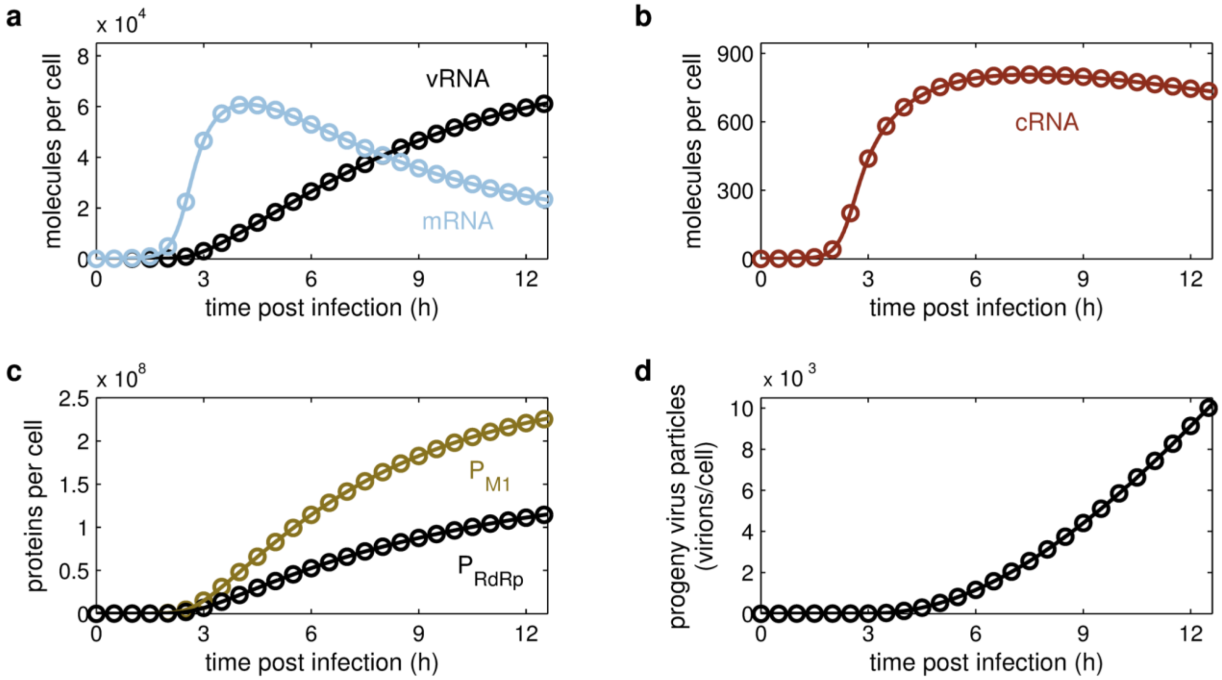

Supplementary Figure 9 | Impact of model modifications in a deterministic setting. The original model of intracellular virus replication[2] (circles) was compared to a deterministic implementation of the model outlined in the Methods section (lines). Results for an infection at an MOI of 10 are shown. (**a**) vRNA and mRNA level of segment 5 (encoding NP). (**b**) cRNA level of segment 5. (**c**) Abundance of unbound viral polymerases (P_{RdRp}) and M1 proteins (P_{M1}). (**d**) Cumulative number of progeny virus particles.

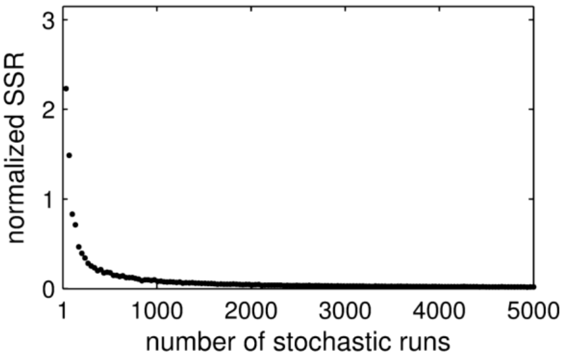

Supplementary Figure 10 | Convergence of mean simulation results with increasing number of runs. The normalized sum of squared residuals (SSR) between the mean of 5000 stochastic runs and the mean of the indicated number of runs is shown for an infection at an MOI of 10. Each dot in the graph corresponds to the average of 500 SSRs calculated by comparing the mean of 5000 stochastic simulations to the mean of the indicated number of simulations, which were drawn randomly from the complete set of runs. The normalized SSRs were calculated over all states, whereby each state was normalized to its maximum across all simulation runs and the number of simulated time points.

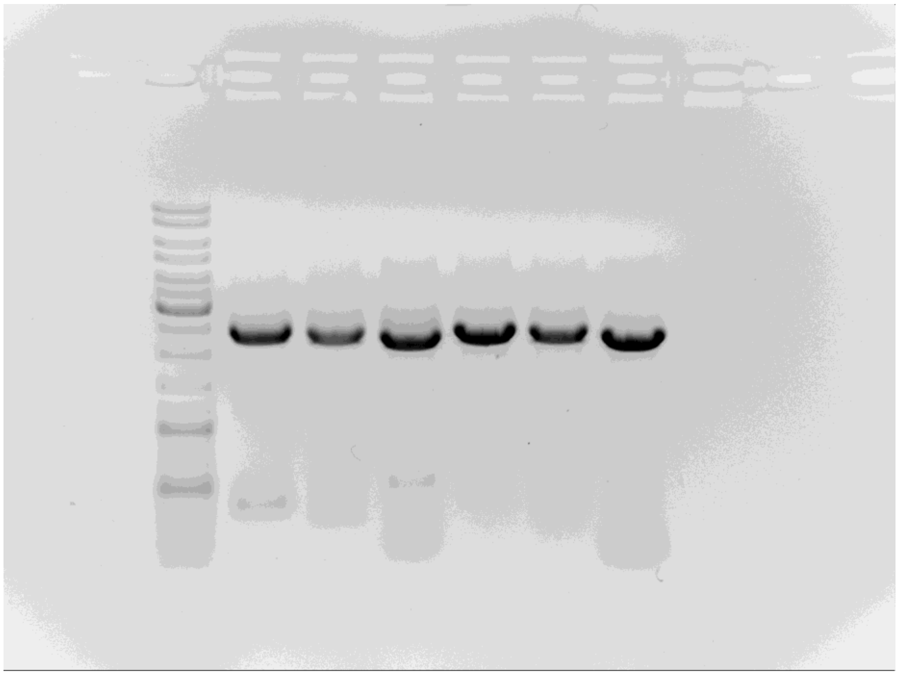

Supplementary Figure 11 | Complete image of the agarose gel shown in Supplementary Figure 6a.

Supplementary Table 1 | Primers utilized for reverse transcription.

Target	Primer name	Sequence (5'-3')	Position (nt)
Segment 3	Seg 3 tagRT for	ATTTAGGTGACACTATAGAAGCGCGAACCGAACGGCTACATTG	731 - 751
Segment 4	Seg 4 tagRT for	ATTTAGGTGACACTATAGAAGCGCCTACTGGTCCTGTTATGT	43 - 62
Segment 5	Seg 5 tagRT for	ATTTAGGTGACACTATAGAAGCGAGTGATTATGAGGGACGGTTGAT	192 - 215
Segment 6	Seg 6 tagRT for	ATTTAGGTGACACTATAGAAGCGTTAGCCATTCAAATTCAAACTG	120 - 141
Segment 7	Seg 7 tagRT for	ATTTAGGTGACACTATAGAAGCGAGCCGAGATCGCACAGAGACTT	87 - 109
Segment 8	Seg 8 tagRT for	ATTTAGGTGACACTATAGAAGCGGATAGTGGAGCGGATTCTG	215 - 234

Supplementary Table 2 | Primers utilized for qPCR.

Target	Primer name	Sequence (5'-3')	Position (nt)
Introduced tag sequence	vRNA tagRealtime for	ATTTAGGTGACACTATAGAAGCG	-
Segment 3	Seg 3 Realtime rev	GAGGCCCATTCGGAAGTCTAAG	825 - 847
Segment 4	Seg 4 Realtime rev	TCTTTCGAATGATGACACTGAG	401 - 423
Segment 5	Seg 5 Realtime rev	CGCACTGGGATGTTCTTC	282 - 300
Segment 6	Seg 6 Realtime rev	CACGGATGGGACAAAGAG	243 - 261
Segment 7	Seg 7 Realtime rev	TGAGCGTGAACACAAATCCTAAAA	201 - 225
Segment 8	Seg 8 Realtime rev	CACTTTCTGCTTGGGTATGA	339 - 359

Supplementary Table 3 | Fractions of vRNA measurements excluded from analysis.

Target vRNA	n_S	Fraction of c_T values beyond standard curve [%]	Fraction w/o c_T value [%]
Segment 3	82	0.00	3.66
Segment 4	82	1.22	1.22
Segment 5	344	8.72	7.85
Segment 6	41	2.44	17.07
Segment 7	123	2.44	1.63
Segment 8	221	9.95	12.67

Table considers data shown in Figure 3a and b, and Figure 5a. n_S indicates number of single cell measurements.

**Supplementary Table 4 | Parameters of the stochastic model.**				
**Parameter**	**Description**	**Value**	**Unit**	**Source**
B_{hi}^{tot}	number of high-affinity binding sites	150	sites	[3]
B_{lo}^{tot}	number of low-affinity binding sites	1000	sites	[3]
D_{Rib}	distance between two adjacent ribosomes	160	nucleotides	[4]
F_{Fus}	fraction of fusion-competent virions	0.51	–	[2]
F_{Spl7}	fraction of M2-encoding mRNAs	0.02	–	based on ratio of M2 to M1 in virion[5]
F_{Spl8}	fraction of NEP-encoding mRNAs	0.125	–	
k_{hi}^{Att}	attachment to high-affinity binding sites	8.09×10^{-2}	$sites^{-1}\cdot h^{-1}$	adjusted to data in reference[3]
k_{lo}^{Att}	attachment to low-affinity binding sites	4.55×10^{-4}	$sites^{-1}\cdot h^{-1}$	adjusted to data in reference[3]
k_{M1}^{Bind}	binding of M1 to nuclear vRNPs	1.39×10^{-6}	$molecule^{-1}\cdot h^{-1}$	[2]
k_{NP}^{Bind}	binding of NP to RdRp-RNA complexes	3.01×10^{-4}	$molecule^{-1}\cdot h^{-1}$	[2]
k_{RdRp}^{Bind}	binding of RdRp-complexes to RNA	1	$molecule^{-1}\cdot h^{-1}$	[2]
k_{M}^{Deg}	degradation of mRNA	0.33	h^{-1}	[2]
k_{R}^{Deg}	degradation of nascent cRNA/vRNA	36.36	h^{-1}	[2]
k_{Rnp}^{Deg}	degradation of RNPs	0.09	h^{-1}	[2]
k_{RRdRp}^{Deg}	degradation of RdRp-RNA complexes	4.25	h^{-1}	[2]
k^{En}	endocytosis	4.8	h^{-1}	[2]
k_{hi}^{Eq}	equilibrium constant of high-affinity sites	1.13×10^{-2}	$sites^{-1}$	[3]
k_{lo}^{Eq}	equilibrium constant of low-affinity sites	8.33×10^{-5}	$sites^{-1}$	[3]
k^{Exp}	NEP binding and nuclear export	1×10^{-6}	$molecule^{-1}\cdot h^{-1}$	adjusted to reference[6]
k^{Fus}	fusion with endosomes	3.21	h^{-1}	[2]
k^{Imp}	nuclear import	6	h^{-1}	[7]
k^{Rel}	virus release	3.7×10^{-3}	$virions\cdot molecule^{-1}\cdot h^{-1}$	[2]
k_{C}^{Syn}	cRNA synthesis	1.38	h^{-1}	[2]
k_{M}^{Syn}	mRNA synthesis	2.5×10^{5}	$nucleotides\cdot h^{-1}$	[2]
k_{P}^{Syn}	protein synthesis	64800	$nucleotides\cdot h^{-1}$	[8]
k_{V}^{Syn}	vRNA synthesis	13.86	h^{-1}	[2]
$K_{V^{Rel}}$	influence of proteins on virus release	10	virions	adjusted
L_{1}^{M}	length of segment 1's mRNA	2320	nucleotides	[9]

L_2^M	length of segment 2's mRNA	2320	nucleotides	9
L_3^M	length of segment 3's mRNA	2211	nucleotides	9
L_4^M	length of segment 4's mRNA	1757	nucleotides	9
L_5^M	length of segment 5's mRNA	1540	nucleotides	9
L_6^M	length of segment 6's mRNA	1392	nucleotides	9
L_7^M	length of segment 7's unspliced mRNA	1005	nucleotides	9
L_8^M	length of segment 8's unspliced mRNA	868	nucleotides	9
L_1^V	length of the vRNA and cRNA of segment 1	2341	nucleotides	9
L_2^V	length of the vRNA and cRNA of segment 2	2341	nucleotides	9
L_3^V	length of the vRNA and cRNA of segment 3	2233	nucleotides	9
L_4^V	length of the vRNA and cRNA of segment 4	1778	nucleotides	9
L_5^V	length of the vRNA and cRNA of segment 5	1565	nucleotides	9
L_6^V	length of the vRNA and cRNA of segment 6	1413	nucleotides	9
L_7^V	length of the vRNA and cRNA of segment 7	1027	nucleotides	9
L_8^V	length of the vRNA and cRNA of segment 8	890	nucleotides	9
$N_{P_{RdRp}}$	number of RdRp-complexes in a virion	45	molecules·virion^{-1}	9
$N_{P_{HA}}$	number of HA molecules in a virion	500	molecules·virion^{-1}	9
$N_{P_{NP}}$	number of NP molecules in a virion	1000	molecules·virion^{-1}	9
$N_{P_{NA}}$	number of NA molecules in a virion	100	molecules·virion^{-1}	9
$N_{P_{M1}}$	number of M1 molecules in a virion	3000	molecules·virion^{-1}	9
$N_{P_{M2}}$	number of M2 molecules in a virion	40	molecules·virion^{-1}	9
$N_{P_{NEP}}$	number of NEP molecules in a virion	165	molecules·virion^{-1}	9
N_{M1}^{Nuc}	nucleotides bound by one M1 molecule	200	nucleotides	10
N_{NEP}^{Nuc}	nucleotides bound by one NEP molecule	1700	nucleotides	adjusted to reference[11]
N_{NP}^{Nuc}	nucleotides bound by one NP molecule	24	nucleotides	11

Supplementary Table 5 | Primer sets for RNA reference standard generation.

Target	Primer name	Sequence (5'-3')	Position (nt)
Segment 3	Seg 3 Uni for	AGCGAAAGCAGGTACTGATCC	1 - 21
	Seg 3 Uni T7 rev	TAATACGACTCACTATAGGGAGTAGAAACAAGGTACTTTTTTGG	2209 - 2233
Segment 4	Seg 4 Uni for	AGCAAAAGCAGGGGAA	1 - 16
	Seg 4 Uni T7 rev	TAATACGACTCACTATAGGGAAGTAGAAACAAGGGTGTTTT	1755 - 1775
Segment 5	Seg 5 Uni for	AGCAAAAGCAGGGTAGATAATC	1 - 22
	Seg 5 Uni T7 rev	TAATACGACTCACTATAGGGAGTAGAAACAAGGGTATTTTTC	1543 - 1565
Segment 6	Seg 6 Uni for	AGCGAAAGCAGGGGTTTAAAATG	1 - 23
	Seg 6 Uni T7 rev	TAATACGACTCACTATAGGGAAGTAGAAACAAGGAGTTTTTTGAAC	1389 - 1413
Segment 7	Seg 7 Uni for	AGCGAAAGCAGGTAGATATTG	1 - 21
	Seg 7 Uni T7 rev	TAATACGACTCACTATAGGGAAGTAGAAACAAGGTAGTTTTTAC	1004 - 1027
Segment 8	Seg 8 Uni for	AGAAAAAGCAGGGTGACAAA	1 - 20
	Seg 8 Uni T7 rev	TAATACGACTCACTATAGGGAAGTAGAAACAAGGGTGTTTT	870 - 890

Supplementary methods

In vitro synthesis of RNA reference standards

Plasmids carrying cDNA of the full-length vRNAs of segment 3 to 8 were utilized in a PCR along with primer sets (Supplementary Table 5) which introduce a T7 promoter sequence in the desired orientation into the PCR products. For this, 5 ng of plasmid was combined with 5 µL of each of the 10 µM primer, 5 µL of a 10 mM dNTP solution, 10 µL of 10 mM $MgCl_2$, 1 x HF buffer, 1 U Phusion High-Fidelity DNA Polymerase and filled to a volume of 100 µL with nuclease-free water (all reagents from Thermo Scientific). The temperature profile comprised denaturation at 98 °C for 3 min, followed by 35 cycles of amplification at 98 °C for 25 s, 53 °C for 45 s and 72 °C for 90 s. Final elongation was performed at 72 °C for 10 min. Purification of the PCR products (InnuPrep PCRpure Kit, Analytik Jena) and subsequent in vitro transcription (TranscriptAid T7 High Yield Transcription Kit, Thermo Scientific) were performed according to the manufacturer's instructions. We then purified the RNA reference standards (NucleoSpin RNA Clean-up, Macherey & Nagel) according to the manufacturer's instructions. Concentration of the in vitro transcribed RNAs was determined spectrophotometrically and RNA integrity was investigated by agarose gel electrophoresis (1 % formaldehyde).

Calculations for absolute quantification

Measurement data of serially 10-fold diluted RNA reference standards was utilized to generate calibration curves using linear regression, whereby c_T values were plotted against $\log_{10}$ numbers of vRNA molecules. Numbers of vRNA molecules ($n_{molecules}$) were

calculated based on the utilized quantity of the corresponding standard dilution (m_{STD} [ng]), the average mass of one base (k=340 [Da/bp]), the length of the fragment ($N_{(bases)}$ [bp]) and the Avogadro constant (N_A [mol^{-1}]).

$$n_{(molecules)} = \frac{m_{STD}}{k \; x \; N_{(bases)} \; x \; 10^9 \; x \; N_A^{-1}}$$

The number of vRNA molecules per single cell (Q_{sample} [vRNAs/cell]) was calculated by considering slope (m) and y-intercept (b) of the calibration curve, F_{RT} (coefficient of dilution of the RT reaction) and V_{lysate} (volume of single cell lysate [µL]).

$$Q_{sample} = 10^{\left(\frac{C_T - b}{m}\right)} \; x \; F_{RT} \; x \; V_{lysate}$$

Supplementary references

1. Frensing T, Pflugmacher A, Bachmann M, Peschel B, Reichl U. Impact of defective interfering particles on virus replication and antiviral host response in cell culture-based influenza vaccine production. *Applied microbiology and biotechnology* 2014, **98**(21)**:** 8999-9008.

2. Heldt FS, Frensing T, Reichl U. Modeling the intracellular dynamics of influenza virus replication to understand the control of viral RNA synthesis. *Journal of virology* 2012, **86**(15)**:** 7806-7817.

3. Nunes-Correia I, Ramalho-Santos J, Nir S, de Lima MCP. Interactions of influenza virus with cultured cells: detailed kinetic modeling of binding and endocytosis. *Biochemistry* 1999, **38**(3)**:** 1095-1101.

4. Arava Y, Wang Y, Storey JD, Liu CL, Brown PO, Herschlag D. Genome-wide analysis of mRNA translation profiles in Saccharomyces cerevisiae. *Proc Natl Acad Sci U S A* 2003, **100**(7)**:** 3889-3894.

5. Robb NC, Jackson D, Vreede FT, Fodor E. Splicing of influenza A virus NS1 mRNA is independent of the viral NS1 protein. *J Gen Virol* 2010, **91**(Pt 9)**:** 2331-2340.

6. Amorim MJ, Bruce EA, Read EKC, Foeglein A, Mahen R, Stuart AD*, et al.* A Rab11 and microtubule dependent mechanism for cytoplasmic transport of influenza A virus vRNA. *J Virol* 2011, **85**(9)**:** 4143-4156.

7. Babcock HP, Chen C, Zhuang X. Using single-particle tracking to study nuclear trafficking of viral genes. *Biophys J* 2004, **87**(4)**:** 2749-2758.

8. Spirin AS. *Ribosome structure and protein biosynthesis*. Benjamin/Cummings Pub. Co., Advanced Book Program: Menlo Park, Calif., 1986.

9. Lamb RA, Krug RM. Orthomyxoviridae: the viruses and their replication. *Fields virology, 4th ed*. p.1487--1531. In D. M. Knipe and P. M. Howley (ed.), Fields virology, 4th ed. Lippincott Williams & Wilkins, Philadelphia, Pa, 2001.

10. Wakefield L, Brownlee GG. RNA-binding properties of influenza A virus matrix protein M1. *Nucleic Acids Res* 1989, **17**(21)**:** 8569-8580.

11. Portela A, Digard P. The influenza virus nucleoprotein: a multifunctional RNA-binding protein pivotal to virus replication. *J Gen Virol* 2002, **83**(Pt 4)**:** 723-734.
